# Hypoxia-Induced Aquaporins and Regulation of Redox Homeostasis by a Trans-Plasma Membrane Electron Transport System in Maize Roots

**DOI:** 10.3390/antiox11050836

**Published:** 2022-04-25

**Authors:** Anne Hofmann, Stefanie Wienkoop, Sabine Lüthje

**Affiliations:** 1 Oxidative Stress and Plant Proteomics Group, Institute of Plant Science and Microbiology, Universität Hamburg, Ohnhorststrasse 18, 22609 Hamburg, Germany; anne.hofmann@uni-hamburg.de; 2 Department of Functional and Evolutionary Ecology, University of Vienna, Djerassiplatz 1, 1090 Vienna, Austria; stefanie.wienkoop@univie.ac.at

**Keywords:** antioxidant, aquaporin, electron transport system, hypoxia, plasma membrane, plant growth regulators, redox homeostasis, root, *Zea mays* L.

## Abstract

In plants, flooding-induced oxygen deficiency causes severe stress, leading to growth reduction and yield loss. It is therefore important to understand the molecular mechanisms for adaptation to hypoxia. Aquaporins at the plasma membrane play a crucial role in water uptake. However, their role during hypoxia and membrane redox changes is still not fully understood. The influence of 24 h hypoxia induction on hydroponically grown maize (*Zea mays* L.) was investigated using an oil-based setup. Analyses of physiological parameters revealed typical flooding symptoms such as increased ethylene and H_2_O_2_ levels, an increased alcohol dehydrogenase activity, and an increased redox activity at the plasma membrane along with decreased oxygen of the medium. Transcriptomic analysis and shotgun proteomics of plasma membranes and soluble fractions were performed to determine alterations in maize roots. RNA-sequencing data confirmed the upregulation of genes involved in anaerobic metabolism, biosynthesis of the phytohormone ethylene, and its receptors. Transcripts of several antioxidative systems and other oxidoreductases were regulated. Mass spectrometry analysis of the plasma membrane proteome revealed alterations in redox systems and an increased abundance of aquaporins. Here, we discuss the importance of plasma membrane aquaporins and redox systems in hypoxia stress response, including the regulation of plant growth and redox homeostasis.

## 1. Introduction

Flooding is a major global problem and occurs more often due to climate change. Flooding induces low-oxygen environments (hypoxia, anoxia), leading to reduced oxygen uptake and cell respiration in the roots. Contrarily, increasing levels of CO_2_ and ethylene were observed [1,2,3] whereby an increase in ethylene is also caused by an upregulation of its biosynthesis [4]. Associated with hypoxia is the overproduction of reactive oxygen species (ROS) that causes lipid peroxidation, protein and nucleic acid oxidation, and, furthermore, cell death [5]. Stress adaptations of plants that are tolerant or resistant to submergence generated two survival strategies: (i) The “low-oxygen escape strategy” (LOES) facilitates oxygen supply by an ethylene-induced shoot elongation, which is believed to be more efficient under shallow long-term flooding. (ii) The “low-oxygen quiescence strategy” (LOQS) maintains energy consumption without shoot elongation which is used by plants coping with deep short-term flooding [6]. Ethylene plays an important role in abiotic stress response. On one side, as part of the LOQS, ethylene triggers the development of adventitious roots [7]. It also stimulates cellulases and pectinases to break down cell walls and therefore form aerenchyma [8,9]. Both adventitious roots and aerenchyma enable the oxygen supply in deficient root zones. On the other side, ethylene triggers an increase in shoot elongation [10,11]. This enables plants to avoid the submergence as part of the LOES. To additionally ensure ATP generation under oxygen deficiency, plants switch to the less efficient anaerobic pathway [11,12,13].

The oxidative stress in low-oxygen environments is a result of an imbalance of ROS production and scavenging [14]. In both, peroxidases (Prx) play a crucial role [15,16,17]. The majority of soluble class III peroxidases were downregulated by hypoxia stress, whereas plasma membrane (PM)-bound peroxidases were induced for membrane protection and cell wall remodeling [18,19]. Other antioxidants such as superoxide dismutase (SOD) and catalase (CAT), as well as thioredoxin (TRX), enzymes of the Foyer–Halliwell–Asada pathway, and L-ascorbate, are involved in the detoxification of ROS [5,20,21,22]. In PM, not only ROS-producing and -scavenging proteins but also transport proteins are located. Aquaporins (AQPs) belong to the major intrinsic proteins (MIPs) [23]. They consist of six transmembrane spanning helices with the N- and C-terminus at the cytosol, five loops, and two highly conserved Asn-Pro-Ala (NPA) motifs [24,25]. Based on sequence similarities, plasma membrane intrinsic proteins (PIPs), as a subgroup of MIPs, are further divided into the subclasses PIP1 and PIP2 [26]. It was shown that PIP1 proteins remain in the endoplasmic reticulum after expression, whereas PIP2 are localized at the PM [27]. The central function of AQPs in plant growth was shown via a four-fold decreased maize root elongation rate after treatment with AQP-inhibiting mercurial compounds [28]. Aquaporins facilitate water transport in either direction [29]. Proteins of the PIP2 subgroup seem to be more efficient as water channels than PIP1 [30,31]. Some PIP1 only act as water channels after forming heterotetramers with PIP2 monomers (e.g., *Zm*PIP1;2 with *Zm*PIP2;1) [27,32]. Studies showed that PIPs can also transport small organic molecules, for example, CO_2_ [31,33,34] or oxygen [23]. Their role in abiotic stress is increasingly studied. The water channel activity is either downregulated by dephosphorylation under drought stress or protonation under flooding stress [35]. The closure of AQPs by protonation of a fully conserved His residue under flooding stress was observed for *So*PIP2;1 [35]. Therefore, regulation of AQP gating by cytosolic pH (acidosis) in anoxic stress has been demonstrated [36]. Evidence for redox regulation was given by oxidative gating of water channels in maize via a decrease in hydraulic conductivity after H_2_O_2_ treatment [37,38]. At least for AQP8, redox regulation by persulfidation of Cys-53 was demonstrated [39]. Aquaporin-8 transports H_2_O_2_ across the PM and is reversibly gated during cell stress, modulating signal strength and duration.

Regarding other PM proteins, physiological functions of the flavocytochrome *b* family, e.g., respiratory burst oxidase homologs (Rboh) in flooding stress and ROS signaling, were explored [40,41]. Furthermore, a constitutive transmembrane redox system, the so-called standard system, was demonstrated in animal and plant PM [42,43,44,45,46]. Evidence for an electron transfer from cytosolic NAD(P)H:quinone oxidoreductases via vitamin K to a cytochrome *b*561 was presented [44,47]. The physiological functions of the cytochrome *b*561 protein family and other PM redox systems still need further elucidation. In addition, the regulation of AQPs and PM redox systems upon hypoxia is not fully understood.

The present study introduced an oil-based hydroponic setup to investigate molecular changes by hypoxia in maize roots using an integrative transcriptomic and proteomic approach. We discuss the importance of AQPs and PM redox systems in hypoxia stress response.

## 2. Materials and Methods

**Figure 1:**
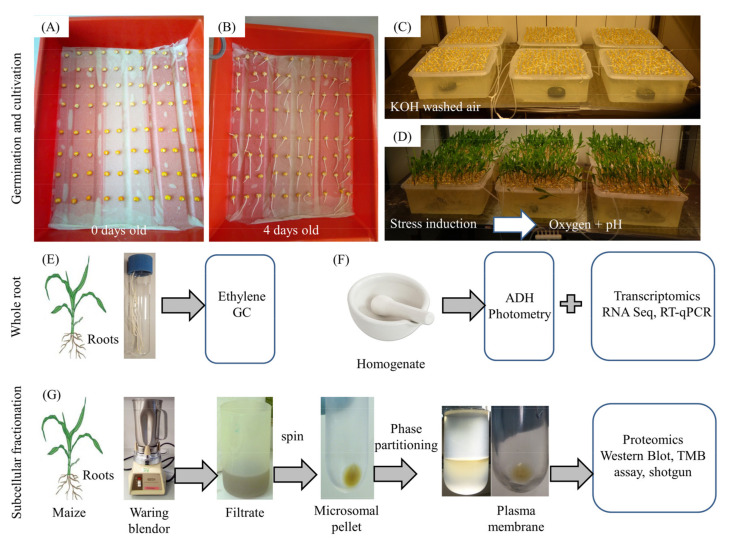
Experimental design. (**A**) Sterilized and soaked caryopses were placed onto wetted tissue and incubated in the dark at 26 °C for four days. (**B**,**C**) The seedlings were transferred to 9 L trays with a culture medium and cultivated in a climate chamber with constant oxygen supply. (**D**) After 14 days, hypoxia stress was induced by adding commercially available rape oil to the culture medium. After 24 h, oxygen and pH were measured and roots of control and stressed plants were sampled. (**E**) Maize roots of control and stressed plants were used to measure ethylene by gas chromatography. (**F**) Maize root extracts of control and stressed plants were used to measure ADH activity and to perform transcriptomic analyses. (**G**) Maize roots of control and stressed plants were used to prepare subcellular fractions (soluble fractions, microsomes, and plasma membranes) for proteomic analysis. For more details, see methods.

Figure 1 shows the steps of the germination and cultivation procedure (Figure 1A–D) as well as the sample preparation for biochemical, transcriptomic, and proteomic analysis (Figure 1E–G).

### 2.1. Plant Material and Growth Conditions

Maize caryopses (*Zea mays* L. cv. Gelber Badischer Landmais, Saatenunion, Hannover, Germany) were cultivated as described before [19]. First, caryopses were sterilized with 3% hydrogen peroxide for 10 min, then washed and soaked in deionized water for at least 4 h with regularly changing the water, then sterilized again. Trays (410 × 300 × 85 mm) were sterilized with 70% ethanol and filled with wetted germination tissue. About 90 caryopses were placed separately onto and covered with wetted tissue. Finally, the trays were covered with aluminum foil for dark incubation at 26 °C for four days. The germinated seedlings were transferred to 9 L plastic boxes filled with hydroponic culture medium (5.25 mM KNO_3_, 7.75 mM Ca(NO_3_)_2_, 4.06 mM MgSO_4_, 1.0 mM KH_2_PO_4_, 100 µM Fe(III)-EDTA, 46 µM H_3_BO_3_, 9.18 µM MnSO_4_, 5.4 µM ZnSO_4_, 9.0 µM CuSO_4_, 2.0 µM Na_2_MoO_4_, pH 5.5) and placed into a climate chamber with a 12 h day–night rhythm (temperature: 22 °C day/18 °C night; light source: Philips SGR 140 with Philips SON-T Agro 400 W sodium vapor lamp, Philips, Hamburg, Germany, 400–500 µmol·m^−2^·s^−1^). The culture medium was replaced after ten days. For 14 days, the medium was oxygenated by KOH-washed air (compressor type LK60, OSAGA, Glandorf, Germany). Then, hypoxia stress was induced by stopping the oxygenation and preventing further oxygen supply by adding 500 mL of commercially available rapeseed oil to the culture medium. This resulted in decreased concentrations of oxygen in the hydroculture medium after 24 h of stress induction (21% to 3.5 ± 0.5% oxygen). Control plants were continuously supplied with air (21% oxygen). For both treatments, the pH thereby stayed stable at 6.8 ± 0.5 to 6.3 ± 0.7 within 24 h. After 24 h, the roots were harvested between 9 and 10 a.m. (CET). Adhered oil was removed from the roots by washing with 0.1% Triton X-100 for 15–30 s and finally with deionized water.

### 2.2. Ethylene and CO_2_

About five maize roots (3–4 g fresh weight) of stressed and non-stressed plants (*n* = 3 biological replicates) were pooled and placed in sealed glass tubes (40 mL volume) for 2 h. With a Hamilton syringe (Chromatographie Service GmbH, Langerwehe, Germany), 500 µL of each gas phase was injected twice (*n* = 2 technical replicates) into a gas chromatograph (Shimadzu GC-14A with C-R5A chromatograph, Kyoto, Japan; 2 m Porapek column with mesh 80–100, column temp.: 50 °C, injector temp.: 80 °C, detector temp.: 80 °C, carrier gas: N_2_ with 30 mL·min^−1^ flow rate, combustion gas: compressed air with 300 mL·min^−1^ flow rate and H_2_ with 30 mL·min^−1^ flow rate). The peak areas for ethylene and CO_2_ were normalized to a reference measurement with an internal standard (10 ppm reference solution in air) and to the fresh weight of the maize roots. Standard deviation and Student’s *t*-test were used to determine significant changes between control and stressed samples.

### 2.3. Alcohol Dehydrogenase (ADH) Activity

About five maize roots (3–4 g fresh weight) of stressed and non-stressed plants (*n* = 3 biological replicates) were pooled and ground in 2 mL of cold homogenization buffer per g fresh weight (50 mM Na_2_HPO_4_ pH 6.8, 5 mM MgCl_2_, 500 µM thiamine pyrophosphate, 5% β-mercaptoethanol, 20% glycerol) [48]. After centrifugation at 34,000× *g* for 15 min at 4 °C (Avanti J-E centrifuge, rotor type JA-25.50, Beckman Coulter, Krefeld, Germany), the clear supernatant was used directly for the assay. For activity measurements of the ADH, 200 µL of this extract was mixed with 0.6 mL 0.1 M pyrophosphate buffer pH 8.0, 100 µL 2 M ethanol, and 100 µL 37.5 mM NAD^+^ to a final volume of 1 mL. The turnover of the substrate NAD^+^ to NADH (ε_340nm_= 6.22 mM^−1^·cm^−1^) was measured three times (*n* = 3 technical replicates) at 340 nm (dual-beam UV/Vis-spectrophotometer, Type UV-1800, Shimadzu, Hamburg, Germany). Standard deviation and Student’s *t*-test were used to determine significant changes between control and stressed samples.

### 2.4. RNA Analysis

#### 2.4.1. RNA Sequencing

RNA isolation was done as described before [19]. The whole root systems, including primary and lateral roots, of five maize plants were harvested after 24 h of hypoxia induction and pooled for each of the three biological replicates (control and stressed), and then ground with liquid nitrogen to a very fine powder (about 0.3 mg fresh weight). This was used for total RNA isolation with the NucleoSpin^®^ RNA Plant and Fungi Kit (Macherey-Nagel, Düren, Germany). For RNA sequence (RNA-Seq) analysis, a final step of ethanol precipitation with a 1/10th volume of 3 M sodium acetate pH 5.2 and 3 volumes of absolute ethanol (>99.5%) was added before delivering the samples to Macrogen Inc. (Seoul, Republic of Korea) for further analyses. Quality control of the samples was performed by Macrogen using agarose gel electrophoresis and an Agilent Technologies 2100 Bioanalyzer (Agilent Technologies, Santa Clara, CA, USA). Six high-quality RNA samples (RNA Integrity Number > 7) were used for cDNA library construction and sequencing using Illumina Sequencing. The following was used as the reference gene: “B73 RefGen_v4”. Available online: https://www.ncbi.nlm.nih.gov/assembly/GCF_000005005.2/ (accessed on 1 April 2019). Expression profiles were represented as the read count and normalized based on the transcript length and depth of coverage. The fragments per kilobase of transcript per million fragments mapped (FPKM) were used for log2 transformation and quantile normalization. Genes with more than 2-fold expression and *p* ≤ 0.05 were considered as differentially expressed genes (DEG).

#### 2.4.2. Quantitative Reverse Transcription Polymerase Chain Reaction (RT-qPCR)

To determine an earlier transcriptional response, nine representative redox and PIP2 proteins with increased abundance in PM after 24 h hypoxia were selected and analyzed after 6 (7.8 ± 1% oxygen) and 12 h (6.4 ± 1.3% oxygen) of treatment using RT-qPCR as described before [19]. The concentration and purity of the isolated total RNA were determined with a Nanodrop spectrophotometer (Fisher Scientific GmbH, Schwerte, Germany). The concentration of total RNA ranged from 200–360 ng·µL^−1^ with an absorbance ratio of 2.13 to 2.19 at 260/280 nm. The cDNA was prepared from 100 ng of total RNA with the First Strand cDNA Synthesis Kit (Fisher Scientific GmbH, Schwerte, Germany) according to the manufacturer´s protocol. A set of primers were designed (Eurofins Genomics Germany GmbH, Ebersberg, Germany, Table 1). Efficiencies of the primers were checked with a 1:2 to 1:200 dilution series by RT-qPCR (5 min 95 °C, 40 cycles of 10 s 95 °C, and 30 s 60 °C terminating in a melting curve from 65 to 95 °C with 0.5 °C s^−1^ steps), using Blue S´Green qPCR Mix Separate ROX (Biozym Scientific GmbH, Hessisch Oldendorf; Germany) and the CFX 96 Cycler (CFX96 Touch system, Bio-Rad, Munich, Germany). The efficiencies of the primers were above 90%. As a housekeeping gene, *Zea mays* translational elongation factor EF-Tu (*zmtufM*, AF264877.1, Q9FUZ6) was used. For RT-qPCR, 1 µg of 1:2 diluted cDNA was used. For statistical analysis, RT-qPCR was performed twice for three biological replicates of each treatment and compared to the housekeeping gene (normalized expression ΔΔCq). Using the CFX manager software version 3.1 (Bio-Rad, Hercules, CA, USA), the expression (relative to zero) was calculated for each stressed sample. Standard deviation and Student´s *t*-test were used to determine significant changes in the expressions.

### 2.5. Preparation of Subcellular Fractions

Subcellular fractions were prepared as described before [19]. Maize roots were washed (3 mM KCl, 0.5 mM CaCl_2_, 0.125 mM MgSO_4_) and homogenized (0.25 M sucrose, 50 mM HEPES, 5 mM Na_2_-EDTA, pH 7.5 supplied with 1 mM dithiothreitol and 1% polyvinylpolypyrrolidone) using a Waring blender 7011HS (Waring, Stamford, CT, USA). The homogenate was filtered through a nylon net (125 µm mesh, Hydro-Bios, Kiel, Germany) and 1 mM phenylmethylsulfonyl fluoride was added. This homogenate was centrifuged (10,000× *g* for 10 min at 4 °C and 48,000× *g* for 30 min at 4 °C, Avanti J-E centrifuge, rotor types JA-14 and JA-25.50, Beckman Coulter, Krefeld, Germany) which resulted in a supernatant with mainly soluble components and a microsomal pellet. Proceeding from the pellet, PMs were isolated with the aqueous polymer two-phase partitioning using 36 g phase systems [49]. Therefore, the pellet was resolved in phase buffer (0.25 M sucrose, 5 mM KCl, 5 mM phosphate buffer, pH 7.8) and applied to the 27 g phase mixture to give a 36 g phase system (0.25 M sucrose, 5 mM phosphate buffer, pH 7.8, 5 mM KCl, 6.5% Dextran T500, 6.5% polyethylene glycol 3350). The phase system was mixed by inverting the tube 20–25 times and then centrifuged at 1,500× *g* for 5 min at 4 °C (Heraeus Christ, Hanau, Germany). The upper phase was transferred to a fresh lower phase and the steps were repeated five times. The final PEG-rich upper phase was centrifuged at 105,000× *g* at 4 °C for 45 min (Optima XPN-80 ultracentrifuge, Beckman Coulter, Krefeld, Germany) and washed twice with buffer (0.25 M sucrose, 50 mM HEPES, pH 7.0) to remove the PEG. The PM pellet was resuspended in this washing buffer and stored at −76 °C. The proteins of the 48,000× *g* soluble fraction were precipitated with 90% saturated (662 g/L) ammonium sulfate at 4 °C overnight, then pelleted at 15,000× *g* at 4 °C for 20 min (Avanti J-E centrifuge, Beckman Coulter, Krefeld, Germany) and resolved (0.25 M sucrose, 50 mM HEPES, pH 7.0) for storage at −76 °C. Proteins were quantified with a modified Bradford assay with 0.01% Triton X-100 using Bovine Serum Albumin Standard (PierceTM, Thermofisher Scientific GmbH, Dreieich, Germany) for calibration [50].

### 2.6. Purity Verification of the PM Fractions

The PMs of the control and 24 h hypoxia-stressed maize root samples were checked for purity using Western blot markers. The washed PM (5 µg) and corresponding microsomes (5 μg) were incubated in 2× reducing loading buffer (32% glycerol, 4% SDS, 0.2 M Tris, 0.16% Bromphenol blue, 5% *β*-mercaptoethanol, pH 6.8) at room temperature for 30 min (H^+^-ATPase) or boiled at 95 °C for 10 min (V-PPase, Cox2), separated on an 11% polyacrylamide gel (10 min 80 V and 90 min 120 V), and transferred to polyvinylidene difluoride membranes (Immobilon PSQ, 0.2 μm pore size, Merck Millipore, Darmstadt, Germany) using transfer buffer (25 mM Tris, 192 mM glycine, 0.01% sodium dodecylsulfate, 10% (*v/v*) methanol, pH 8.3) and semi-dry blotting system (Fastblot B33, Biometra, Analytik Jena Company, Germany) with 30 V and 150 mA for 1 h. Afterward, membranes were blocked with 5% milk (instant skim milk powder, Frema, Herbolzheim, Germany) in TBST (50 mM Tris, 150 mM NaCl, 1% Tween 20, pH 7.6) at room temperature for 30 min and incubated with the first antibody in 5 mL TBST at 4 °C overnight. The following antibodies were purchased from Agrisera (Vännäs, Sweden) and used to detect the PM-specific H^+^-ATPase of plants (H^+^-ATPase, #AS07260, 1:5000), the vacuolar H^+^-pyrophosphatase (V-PPase, #AS121849, 1:2500), and the mitochondrial cytochrome *c* oxidase (Cox2, #AS04053A, 1:1000). Horse radish peroxidase-coupled goat anti-rabbit served as a secondary antibody (#AS09602, 1:25,000) prior to enhanced chemiluminescence (ECL) detection using ECL reagents (HRP-Juice, PJK, Kleinblittersdorf, Germany) and the LAS-3000 Imaging system (Fujifilm, Tokyo, Japan).

### 2.7. Tetramethylbenzidine Oxidase Activity

The activity of *haem* and copper-containing proteins was determined in soluble and PM fractions of maize roots of at least four biological replicates per treatment. For this, a solution with 4.7 mM 3,3′,5,5′-tetramethylbenzidine (TMB, PanReac AppliChem, Darmstadt, Germany), 30% methanol in 50 mM sodium acetate buffer pH 5.0) was prepared prior to use [adapted from 49]. The assay contained 790 µL 25 mM sodium acetate buffer pH 5.0, 100 µL 0.3% H_2_O_2_ (AppliChem, Darmstadt, Germany), 100 µL TMB solution, and 10 µL protein sample with an average of 0.6 µg soluble protein and 4.5 µg PM protein, respectively. The turnover of TMB was measured photometrical (UV-1800 spectrophotometer, Shimadzu Cooperation, Kyoto, Japan) for 2 min at 652 nm (ε_652 nm_ = 39 mM^−1^·cm^−1^). The buffer and the two substrates served as a reference. Standard deviation and Student´s *t*-test were used to determine significant changes between control and stressed samples.

### 2.8. Shotgun Proteomics of the Plasma Membrane and Soluble Fraction

Sample preparation (at least three biological and two technical replicates of control and stressed samples) and further mass spectrometry (MS) analysis were done as described before [19]. First, PMs (100 µg) were washed in 2 mL (250 mM sucrose, 50 mM HEPES, 150 mM KCl, 0.01% Triton X-100) for 30 min, then pelleted for 1 h at 16,060× *g* at 4 °C (Biofuge Fresco, Heraeus, Hanau, Germany). The pellets were incubated in 200 µL solubilization buffer (125 mM Tris pH 6.5, 2% sodium dodecyl sulfate, 5% *β*-mercaptoethanol, 6 M urea) for 1 h at room temperature and centrifuged again at 16,060× *g* at 4 °C for 60 min. Proteins of the resulting supernatant (PMs) and proteins of the soluble 48,000 g fraction (100 µg) were precipitated with 1.8 mL methanol:chloroform (4:1) at −20 °C overnight, centrifuged, and washed three times in 0.5 mL pure methanol with centrifugation at 16,060× *g* at 4 °C for 20 min. The pellets were air-dried and digested in 50 µL buffer (200 mM NH_4_CO_3_ pH 8.5, 8 M urea, 10% acetonitrile) with 0.2 µg lysine C (New England Biolabs GmbH, Frankfurt am Main, Germany) at 37 °C for 16–18 h. Then, the samples were diluted 1:3 in 10% acetonitrile, and 10 µL trypsin beads (Poroszyme bulk immobilized trypsin, Applied Biosystem, Waltham, USA) were added and incubated at 37 °C for 16–24 h. The digestion was stopped by adding three times the volume of 0.3% heptafluorobutyric acid and the trypsin beads were removed by multiple centrifugations. Peptides were washed and dried using ZipTips (Agilent Technologies, Santa Clara, CA, USA) in accordance with the manufacturer´s protocol.

The peptides were dissolved in 2% acetonitrile and 0.1% formic acid. For each sample, 1 µg was applied randomly on a C18 column (15 cm, 50 mm, column, PepMapR RSLC, 2 µm particle size, Thermo Scientific, Bremen, Germany) for separation during a 90 min gradient at a flow rate of 300 nL min^−1^. Measurements were done on a QExactive (Thermo Fisher Scientific, Bremen, Germany) with the following settings: full scan range 350–1800 *m*/*z*, max 20 MS2 scans (activation type CID), repeat count 1, repeat duration 30 s, exclusion list size 500, exclusion duration 60 s, charge state screening enabled with a rejection of unassigned and +1 charge states, minimum signal threshold 500. Proteins were identified and quantified using a UniprotKB FASTA download for *Zea mays* (UP000007305, downloaded July 2017 for PM and downloaded November 2021 for soluble fraction) and the software MaxQuant v1.6.5.0 with the following parameters: first search peptide tolerance 20 ppm, main search tolerance 4.5 ppm, ITMS MS/MS match tolerance 0.6 Da. A maximum of three of the following two variable modifications were allowed per peptide: oxidation of methionine and acetylation of the N-term. A maximum of two dismissed cleavages were tolerated. The best retention time alignment function was determined in a 20 min window. Identifications were matched between runs in a 0.7 min window. An FDR cutout at 0.01 (at Peptide Spectrum Match and protein level) was set with a reversed decoy database. A minimum of seven amino acids was required for the identification of peptides and at least two peptides were required for protein identification. The resulting data matrix was filtered so that there are label-free quantification (LFQs) values in more than four replicates (biological and/or technical replicates) of at least one of the treatments. Missing values were corrected with COVAIN [51]. The LFQ intensities of the replicates were averaged for statistical analyses. Standard deviation and Student´s *t*-test were used to determine significant changes between control and stressed samples.

### 2.9. Analyses of Shotgun Proteomics of the PMs and RNA Sequencing Data

Using the UniProt accessions of the shotgun proteomics data, the gene identification numbers (Entrez_Gene_IDs) were identified. Gene-set enrichment (GO) analyses of the RNA-Seq and shotgun data of the PMs were performed using DAVID v6.8 (for Database for Annotation, Visualization and Integrated Discovery; Available online: https://david.ncifcrf.gov/ (accessed on 2 July 2021) [52,53] with a Fisher´s test and Bonferroni correction related to the categories “biological process”, “molecular function”, and “cellular component”. DAVID tool also provides the KEGG (for Kyoto Encyclopedia of Genes and Genomes) pathway, INTERPRO, and SMART domains (for Simple Modular Architecture Research Tool). Prediction of the transmembrane helices was performed with TMHMM Server v2.0 (Available online: http://www.cbs.dtu.dk/services/TMHMM/ (accessed on 3 June 2021)) [54]. Protein parameters (molecular weight and pI) were predicted with ProtParam (Available online: https://web.expasy.org/protparam/ (accessed on 3 June 2021)) [55]. Promoter analyses were done with NewPLACE (Available online: https://www.dna.affrc.go.jp/PLACE/?action=newplace (accessed on 3 June 2021)) [56]. Expression profiles that were obtained from the NimbleGen microarray, provided by [57], were extracted from the Maize eFP Browser.

The proteomics data are available via ProteomeXchange with identifiers PXD028987 (PM) and PXD033386 (soluble fraction).

## 3. Results

### 3.1. Ethylene Induction and Metabolic Switch as an Indicator for Hypoxia Stress

**Figure 2:**
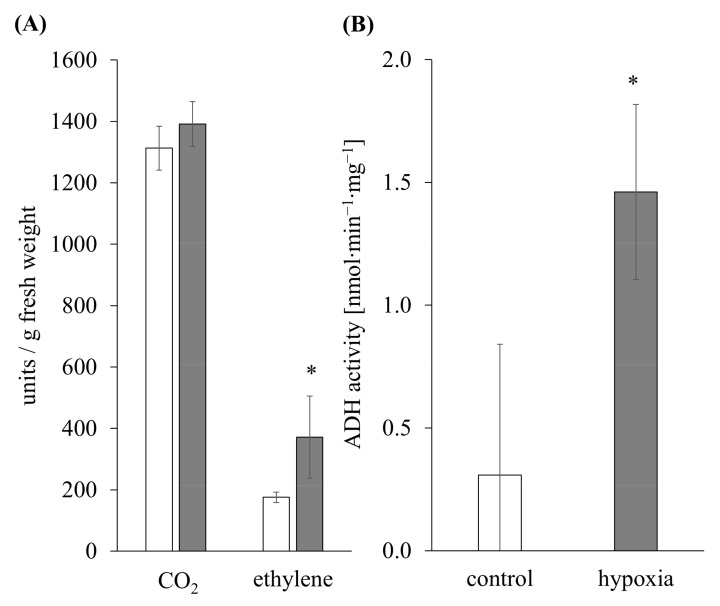
Physiological changes of maize plants under 24 h of hypoxia. (**A**) CO_2_ and the gaseous plant hormone ethylene were analyzed in control (white columns) and 24 h hypoxia-stressed maize roots (gray columns) using a Shimadzu GC-14A gas chromatograph for three biological and two technical replicates per sample. The amounts were normalized to the fresh weight of the roots. Error bars indicate standard deviation. Significances are indicated with asterisk (*p* < 0.05 *) determined with Student´s *t*-test (*n* = 3). (**B**) The activities of the alcohol dehydrogenase (ADH) were determined three times for three biological replicates of control and 24 h hypoxia-stressed maize roots. Error bars indicate standard deviation. Significances are indicated with asterisk (*p* < 0.05 *) determined with Student´s *t*-test (*n* = 3).

Maize roots, stressed with hypoxia for 24 h, showed no difference in the CO_2_ amount compared to the non-stressed plants (controls 1313 ± 72 units/g fresh weight; stressed 1391 ± 73 units/g fresh weight). Instead, the amount of ethylene increased significantly by 2-fold (controls 175 ± 17 units/g fresh weight; stressed 371 ± 134 units/g fresh weight) (Figure 2A). Total extracts of 24 h hypoxia-stressed maize roots showed a significantly 4-fold increased activity of ADH (1.46 ± 0.35 nmol·min^−1^·mg^−1^) compared to the controls (0.30 ± 0.53 nmol·min^−1^·mg^−1^) (Figure 2B).

RNA sequence analysis determined the genes of the ethylene biosynthesis pathway and receptors (Supplementary Table S1) that contained five 1-aminocyclopropane-1-carboxylate (acc) synthase genes with one more than 2-fold upregulated (gene ID 100217270), 16 acc oxidase genes with one 2-fold downregulated (gene ID 100280566), and four more than 2-fold upregulated (gene IDs 103641391, 100283053, 100273458, and 542136). Three of them were differentially expressed (103641391, 542136, and 100273458). Eight ethylene receptor genes were found, which were mainly non- or slightly downregulated. Only one of them, *ethylene receptor homolog 2* (*zmetr2*, gene ID 541627), showed a more than 2-fold increase.

Genes of the anaerobic pathway (ldh, pdc, adh) were found in RNA-Seq analysis (Supplementary Table S1). Two of the four ldh genes showed a more than 2-fold increase (gene IDs 100193279 and 100282503). The three pdc genes were all more than 5-fold upregulated, with one of them even differentially expressed (gene ID 542651). Fourteen adh genes were found, of which two genes were 6- and 9-fold upregulated (*Zmadh1*, gene ID 542363 and *Zmadh2*, gene ID 542364). Two putative adh superfamily genes were differentially downregulated by 4.5- and 2.8-fold (gene IDs 100383565 and 100272357), respectively.

The *cytochrome bc1 complex* and the *cytochrome oxidase 2* are key enzymes of the aerobic pathway. The four found genes were all downregulated, one significantly by 1.8-fold (gene ID 100383274). An alternative oxidase was not regulated on the transcript level (Supplementary Table S1). In the shotgun data of the soluble fraction, one ADH protein (*Zm*ADH, A0A1D6GEX5) was found with a 5-fold decreased abundance (Supplementary Table S2).

### 3.2. RNA Sequence Analysis

**Figure 3:**
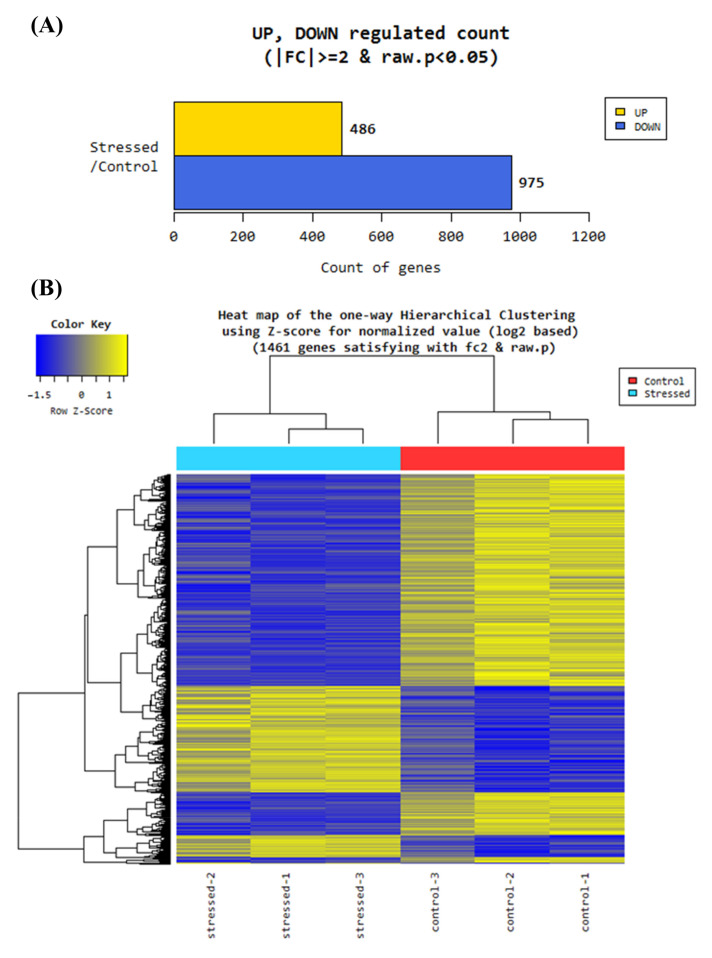
Hierarchal clustering of differentially expressed genes in maize roots induced by 24 h hypoxia. (**A**) In RNA-Seq analysis of maize roots under 24 h of hypoxia, 29,703 normalized genes were analyzed, of which 486 were differentially up- and 975 were differentially downregulated based on 2-fold change and *p* < 0.05. (**B**) The heat map of the hierarchical clustering of the differentially expressed genes revealed clusters of up- and downregulated genes.

The quality control report of the RNA-Seq analysis revealed more than 90% of the bases with quality over a Phred score Q30 for each sample, where the base call accuracy is 99.9% (Supplementary Figure S1). In total, 49,607 genes were determined. After quantile normalization, 29,703 genes were analyzed regarding up- or downregulation. From these normalzsed genes, 1461 genes were differentially expressed (DEG; 975 down- and 486 upregulated) based on 2-fold change and a *p*-value of *p* < 0.05 of the comparison pair (stressed versus control) (Figure 3A). The hierarchical clustering of the DEGs is shown in the heat map (Figure 3B).

#### 3.2.1. Gene Ontology Analysis of Differentially Regulated Genes

**Figure 4:**
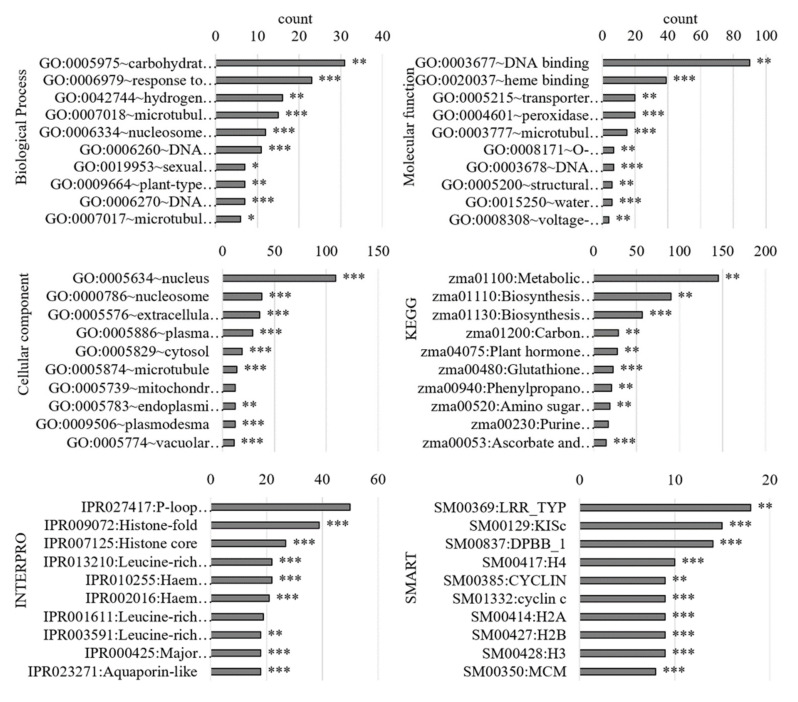
Gene-set enrichment analysis of maize roots under 24 h of hypoxia using DAVID. Differentially expressed genes were used to perform gene-set enrichment (GO) analyses with DAVID regarding the GO terms “biological process”, “molecular function”, and “cellular component”. In addition, KEGG pathways and domain analyses with INTERPRO and SMART were performed. The top 10 terms of each category with the corresponding number of genes (count) are shown in the graph with the *p*-values as asterisks (*p* < 0.05 *, *p* < 0.01 **, *p* < 0.001 ***). LRR_TYP, Leucine-rich repeats, typical subfamily; KISc, Kinesin motor, catalytic domain. ATPase; DPBB_1, Rare lipoprotein A (RlpA)-like double-psi beta-barrel; H4, Histone H4; CYCLIN, domain present in cyclins, TFIIB, and Retinoblastoma; H2A, Histone 2A; H2B, Histone H2B; H3, Histone H3; MCM, minichromosome maintenance proteins.

Gene ontology analysis with DAVID revealed that the DEGs were only partially mapped (Supplementary Table S3A). The top 10 terms of each category for all DEGs are shown in the graph (Figure 4, Supplementary Table S4A–C). The main biological processes were “response to oxidative stress” (GO:0006979), “hydrogen peroxide catabolic process” (GO:0042744), and “plant-type cell wall organisation” (GO:0006270), which indicated stress adaptations. The main molecular functions were “heme binding” (GO:0020037), “transporter activity” (GO:0005215), “peroxidase activity” (GO:0004601), and “water channel activity” (GO:0015250), which were also related to these processes. The nucleus (GO:0005634) was the main cellular component, followed by the extracellular region, PM, and cytosol. In addition, the main domains found with INTERPRO were *haem* peroxidase (IPR010255, IPR002016) and AQP-related (IPR000425, IPR023271). Other domains regarding histones were determined with INTERPRO and SMART. The main domain in SMART was Leucine-rich repeats (LRR).

#### 3.2.2. RNA Sequence Analysis of Genes Related to Redox System, Antioxidant Biosynthesis, and Transporters

With RNA-Seq, the expression of redox system-related genes were analyzed (Supplementary Table S1). From the *haem* peroxidases, genes of 139 class III peroxidases, 10 ascorbate peroxidases (apx), two cat, and one dioxygenase (diox) were identified. Of those genes, differentially upregulation was determined for *zmprx06*, *zmprx117*, and *zmprx135* (gene ID 100383323, 103640022, and 103635633), *zmapx04*, and *zmdiox01*. Differentially downregulation was observed for 14 class III peroxidases, *zmapx03* and *zmapx05* (gene IDs 100282326 and 103639279), and *zmcat2* (gene ID 542230). From the non-*haem* peroxidases, five glutathione peroxidase genes (gpx) and peroxiredoxins including one atypical 2 Cysteine peroxiredoxin gene (type Q, prxq), three atypical 2 Cysteine peroxiredoxins genes (type II, prxII), two typical 2 Cysteine peroxiredoxins genes (2cysprx), and one Cysteine peroxiredoxin (1cysprx) were identified. Only the peroxiredoxin *zm2cysprx02* was 3-fold differentially downregulated. From other oxidoreductases, genes of 12 rboh, 5 sod, 3 disulfide isomerases (pdil), 1 trx, 4 glutathione reductases (gr), 4 dehydroascorbate reductases (dhar), 4 monodehydroascorbate reductases (mdhar), 3 ascorbate oxidases (ao), 13 lipoxygenases (lox), 4 auxin-induced in root cultures (air12), 6 monocopper oxidase-like proteins (sku5), 23 cytochromes, 12 malate dehydrogenases (mdh), and 5 NAD(P)H:quinone oxidoreductases (tryptophane repressor binding protein A; wrba) have been identified. Differentially upregulated was *lipoxygenase 4* (*zmlox4*, gene ID 100037803). Differentially, downregulation was determined for protein *disulfide isomerase 2* (*zmpdil1-2*, gene ID 606410), *monodehydroascorbate reductase 5* (*zmmdhar5*, gene ID 100272772), *lipoxygenase 12* (*zmlox12*, gene ID 100037829), *air12* (*zmair12*, gene ID 100285927), *sku5* (*zmsku5*, gene ID 100383470), *cytochrome b561 and DOMON domain-containing protein* (DOMON for dopamine β-monooxygenase; *zmDoHcytb561*, gene ID 100191661), two mdh genes (gene IDs 100272900 and 100273428), and two wrba genes (gene IDs 100283201 and 100285601).

Regarding vitamin K_1_ biosynthesis, eight genes of the three pathways (menaquinone, phylloquinone, and ubiquinone pathway) were found in RNA-Seq analysis. The *demethylmenaquinone methyltransferase* (*menG*, gene ID 100284521), which produces menaquinol, was significantly 1.73-fold upregulated. The *isochorismate synthase* (*menF*, gene ID 100275708), the first protein in the phylloquinone pathway, was significantly more than 2-fold downregulated. Contrary, the *1,4-dihydroxy-2-naphthoyl-CoA synthase* (*menB*, gene id 100304279) and the *demethylphylloquinol methyltransferase* (gene ID 100384459), the proteins that produce vitamin K_1_, were not regulated. The *3-demethylubiquinol 3-O-methyltransferase* (gene ID 100272292) of the ubiquinone biosynthetic pathway was significantly 1.55-fold upregulated.

Regarding the L-ascorbate biosynthesis pathway, seven genes were found including the last two enzymes *L-galactono-1,4-lactone dehydrogenase* (gene ID 100381436) and *L-galactose dehydrogenase* (gene ID 732776) which were downregulated. In addition, *GDP-D-mannose 3′,5′-epimerase* (gene IDs 100192102 and 100280325) and one of the three found *phosphomannomutases* (gene ID 100272867) were downregulated.

Genes of seven nucleobase-ascorbate transporters were either not or downregulated. Genes of 13 Casparian strip-like proteins (CASPL) were found, with 3 more than 2-fold downregulated (gene IDs 100281882, 100282577, and 100285037) and 1 upregulated (gene ID 100285613). Genes of six aluminum activated malate transporters (ALMTs) were found in RNA-Seq analysis, with one (gene ID 103627532) significantly 1.55-fold upregulated. Eleven genes of the receptor-like kinases (RLK) were found, with two that were upregulated.

The expression of 35 aquaporin genes, including pips, tonoplast intrinsic proteins, nodulin26-like intrinsic proteins, and small basic intrinsic proteins, were determined. Almost all maize PIPs decreased significantly by 2-fold, excluding *zmpip1;5*, *zmpip2;1*, and *zmpip2;2* where the gene expression showed no significant decrease between controls and hypoxia-stressed samples (Table 2). The gene expression of *zmpip2;7* was not detected.

#### 3.2.3. Gene Expression and Promotor Analysis of PM-Bound Redox Systems and PIPs

**Figure 5:**
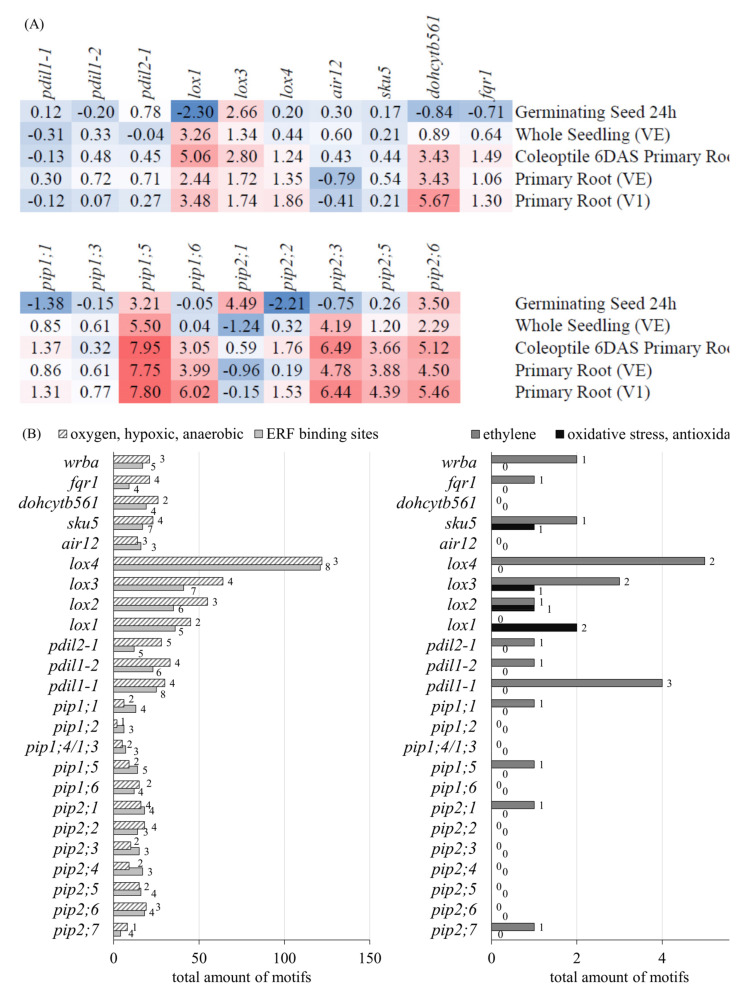
Analysis of PM-bound redox system and PIPs in maize roots. (**A**) Heat map of expression profiles in log2 ratios. Expression profiles, were extracted from Maize eFP Browser. (**B**) Promoter motifs were clustered regarding the keywords anaerobic, hypoxic, and oxygen (shaded bars), binding sites of ethylene-responsive factors including ERE, GCC box, and DRE (light gray bars), ethylene (dark gray bars), and oxidative stress, antioxidants (black bars). The total copy number of all motifs within these three groups is shown in the graph. The bars are labeled with the amount of the different motifs.

The gene expression of PM-bound proteins revealed a relative strong expression in primary roots for *zmlox1*, *zmlox3*, *zmlox4*, *zmfqr1*, and *zmdohcytb561* (Figure 5A). The minimal expression was determined for *zmair12*, the maximal expression for *zmdohcytb561*. In addition, *pips* showed a relative strong expression in primary roots. The minimal expression was determined for *zmpip2;1*, the maximal expression for *zmpip1;5*.

The analyses of *cis*-regulatory elements (Figure 5B) concluded a regulation by abiotic factors (anaerobic, hypoxia, and oxygen) including all *pips*. In addition, motifs for binding sites of ethylene responsive factors (ERF) were found (GCC box, ERE, and DRE), in spite of the fact that the motif for the hormonal regulator ethylene was not determined for *zmdohcytb561*, *zmair1*2, and *zmlox1* genes, and only in four of the 12 *pips* (*zmpip1;1*, *zmpip1;5*, *zmpip2;1*, and *zmpip2;7*). A motif found in *Arabidopsis thaliana* ADH gene promoter (Adh; S000133; CCACGTGG) was detected in *zm*pdil1-1 and *zm*pdil1-2, *zm*lox4, and *zm*sku5 and for *zmpip1;5* only. Motifs regarding oxidative stress were determined in *zmlox1*, *zmlox2*, *zmlox3*, and *zmsku5*, but not for the *pips*.

#### 3.2.4. RT-qPCR

**Figure 6:**
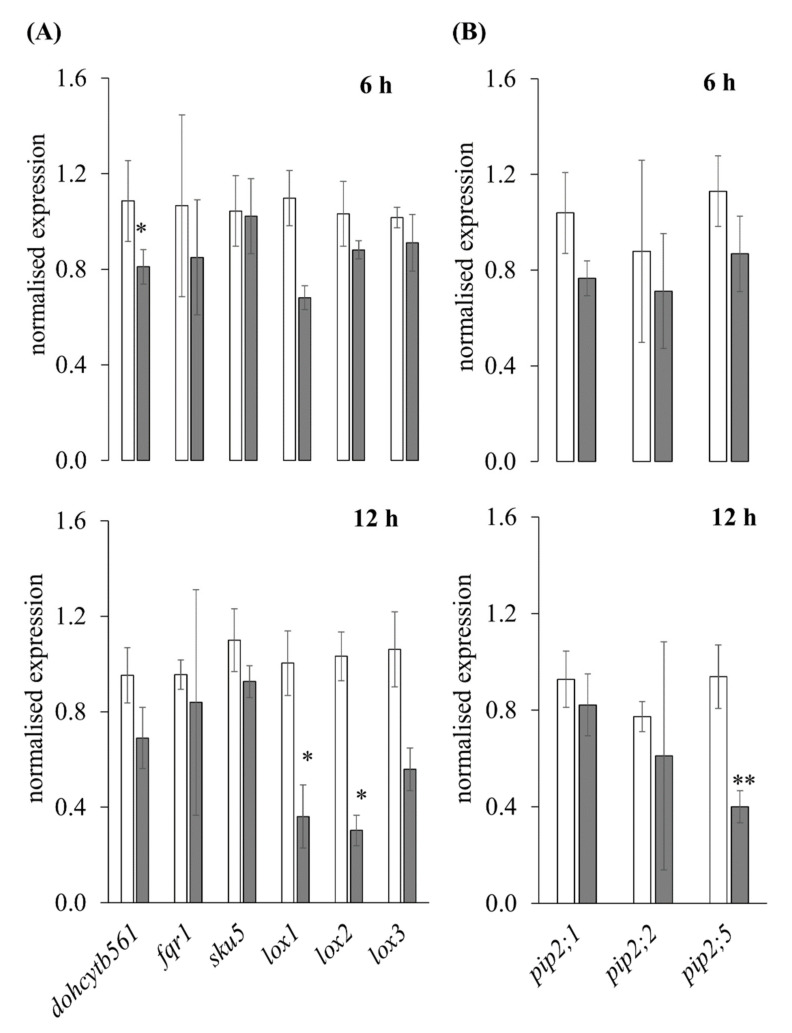
Regulation of PM-bound redox systems and PIPs by hypoxia stress. Expression analysis of selected genes using SYBRGreen RT-qPCR with total RNA of three non-stressed (white columns) and three hypoxia-stressed (dark gray columns) maize roots either after 6 or 12 h after treatment. Shown are (**A**) six redox system-related genes and (**B**) three representative *pip* genes. Expression was normalized to *zmtufM* as a housekeeping gene using Biometra CFX96 software. Significances were calculated with Student´s *t*-test and marked with *p* < 0.05 * and *p* <0.01 ** for three biological and two technical replicates per sample.

The expression analysis with RT-qPCR (Figure 6) of 6 and 12 h hypoxia-stressed samples determined a downregulation of the six selected genes *zmdohcytb561* (1.34- and 1.38-fold), *zmfqr1* (1.25- and 1.14-fold), *zmsku5* (1.05- and 1.19-fold), *zmlox1* (1.61- and 2.78-fold), *zmlox2* (1.17- and 3.41-fold), and *zmlox3* (1.12- and 1.90-fold). Additionally, three selected aquaporin genes *zmpip2;1* (1.36- and 1.13-fold), *zmpip2;2* (1.23- and 1.27-fold), and *zmpip2;5* (1.30- and 2.35-fold) were downregulated.

### 3.3. Shotgun Analysis

**Figure 7:**
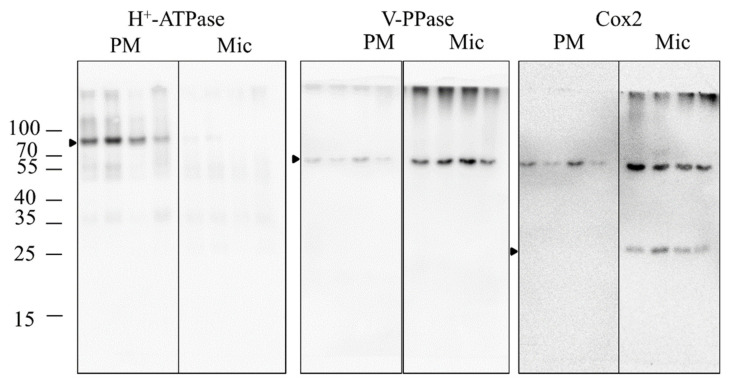
Purity verification of PM preparations. Plasma membrane and corresponding microsomal protein samples (5 µg each) were load on 11 % polyacrylamide gels and transferred to PVDF membrane. Following antibodies were used to detect the PM specific H^+^-ATPase of plants (H^+^-ATPase, 1:5000), the vacuolar H^+^-pyrophosphatase (V-PPase, 1:2500), and the mitochondrial cytochrome *c* oxidase (Cox2, 1:1000). Shown are two representative samples.

In the shotgun analysis of the PMs, a total of 485 proteins were analyzed, of which 165 were filtered out (see methods). Data are available via ProteomeXchange. Protein immunoblots of the PMs showed an enrichment of the H^+^-ATPase and a lower amount of V-PPase and Cox2 signals compared to the corresponding microsomal fractions (Figure 7). In addition, marker analyses of the shotgun data revealed highly enriched PM preparations (Supplementary Table S5A). Plasma membrane-specific P-type ATPases (K7TX67, A0A1D6MV33, and A0A1D6DVJ7) were found in all PM samples, but with higher amounts in stressed samples. Vacuolar V-ATPases (C0PHC0, B6UHI4, and A0A1D6JW70) were detected but with less amount than H^+^-ATPase and a reduction in stressed samples. Vacuolar V-PPases and mitochondrial cytochrome *c* oxidase were not detected. Proof of luminal-binding protein 2 BiP2 (P24067) and calreticulin-2 (A0A1D6EN43) suggested typical contamination by the endoplasmic reticulum. The Golgi marker GTP-binding protein SAR1 (B7ZZP2), but not the Sec21p protein, was detected. Typical cell wall proteins (expansins) were not found. Cytosolic markers such as ADH, glyceraldehyde-3-phosphate dehydrogenase, or fructose-1,6-bisphosphatase were not observed in PM samples.

#### 3.3.1. Gene Ontology Analysis of the PM-Bound Proteins

**Figure 8:**
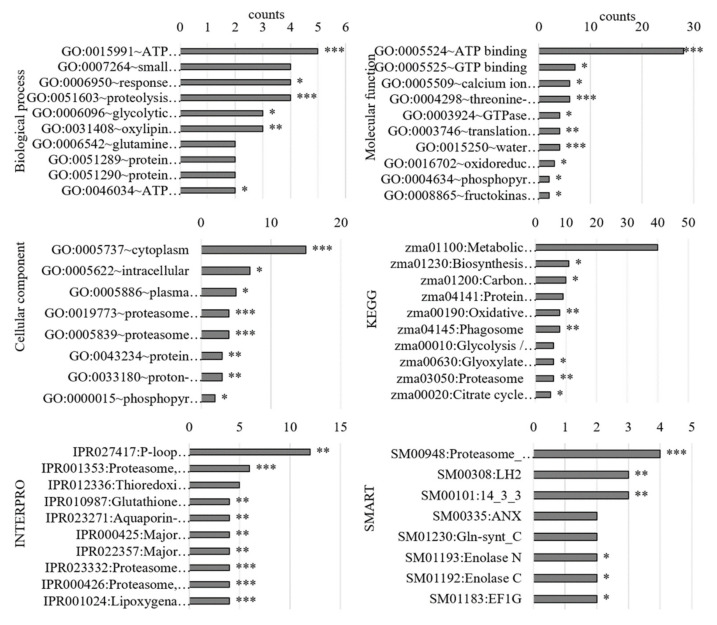
Gene ontology analyses of PM-bound proteins altered by hypoxia. Plasma membrane-bound proteins that were significantly altered by 24 h of hypoxia were determined by mass spectrometry (shotgun). Using DAVID, gene ontology (GO) enrichment analyses were performed. The top five GO terms and the corresponding number of genes are shown in the graph. Asterisks indicate *p*-values (*p* < 0.05 *, *p* < 0.01 **, *p* < 0.001 ***). ANX, annexin repeats; Proteasome_A_N, proteasome subunit A N-terminal signature; LH2, lipoxygenase homology 2 (beta barrel) domain; 14_3_3, 14-3-3 homologues; Gln-synt_C, glutamine synthetase, catalytic domain; Enolase N, enolase, N-terminal domain; Enolase C, enolase, C-terminal TIM barrel domain; EF1G, elongation factor 1 gamma, conserved domain.

Gene ontology analysis with DAVID revealed that the genes of the altered proteins were only partially mapped (Supplementary Table S3B). The top 10 terms of each category for the genes of all proteins are shown in the graph (Figure 8, Supplementary Table S6A). DAVID analysis of the proteins with increased abundances in PM after 24 h of hypoxia (Supplementary Table S6B) were categorized regarding (i) the biological process in the “hydrogen peroxide catabolic process” (GO:0042744) and “response to oxidative stress” (GO:0006979), (ii) the molecular function in “water channel activity” (GO:0015250), “transporter activity” (GO:0005215), “oxidoreductase activity” (GO:0016702), and “peroxidase activity” (GO:0004601), and (iii) the cellular component in “plasma membrane” (GO:0005886). KEGG analysis revealed “metabolic pathways” (zma01100) such as “Biosynthesis of secondary metabolites” (zma01110). The INTERPRO and SMART domains that were found were related to redox proteins such as lipoxygenase (IPR001024, IPR020833, IPR001246, IPR000907, IPR019794, and SM00308), aquaporins (IPR022357, 000425, and 023271), and peroxidases (IPR019794). Proteins with decreased abundances in the PM (Supplementary Table S6C) were categorized in mainly “lytical processes” (GO:0051603, GO:0015991, and GO:0006096) and “ATP and GTP bindings” (GO:0005524 and GO:0005525). “Protein processing pathway in the ER” (zma04141) and “biosynthesis of amino acids” (zma01230) were the major KEGG pathways. “Proteasome domains” (IPR001353, IPR000426, IPR023332, and SM00948) and “14-3-3 kinase domains” (IPR023409, IPR023410, IPR000308, and SM00101) were found to be the main domains.

#### 3.3.2. TMB Oxidation

**Figure 9:**
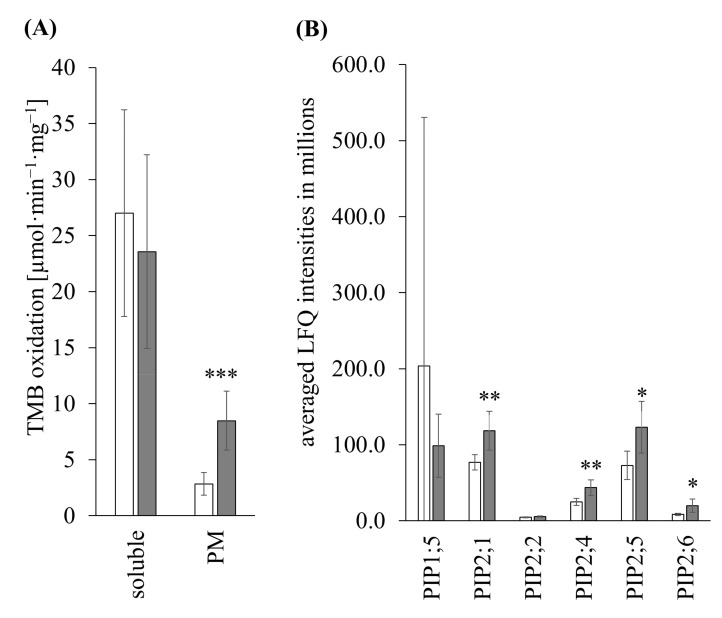
Alteration of PM-bound *haem* proteins and PIPs. (**A**) For maize root soluble proteins and PMs, the activities of *haem* and copper-containing proteins were determined spectrophotometrically using the substrates H_2_O_2_ and 3,3′,5,5′-tetramethylbenzidine (TMB). (**B**) Plasma membranes of control and 24 h hypoxia-stressed maize roots were used for mass spectrometry analyses. Label-free quantification (LFQ) data were determined with MaxQuant software and used for further statistical analyses. Control (white columns) and stressed samples (gray columns) were shown. Error bars indicate standard deviation. Significances are indicated with asterisk (*p* < 0.05 *, *p* < 0.01 **, *p* < 0.001 ***) determined with Student´s *t*-test for TMB oxidation (*n* > 4 biological replicates and *n* = 3 technical replicates) and for shotgun (*n* = 3 biological and *n* = 2 technical replicates per treatment).

Focusing on the subcellular fractions of maize roots, the TMB oxidase activity of putative *haem* and copper-containing proteins in the soluble fraction decreased from 27.00 ± 9.22 in the control to 23.56 ± 8.64 µmol·min^−1^·mg^−1^ in stressed samples (Figure 9A). This was a decrease to 87% activity in stressed samples relative to the controls. In the PM fraction, the activity increased significantly by 2.9-fold from 2.84 ± 1.02 in control samples to 8.48 ± 2.64 µmol·min^−1^·mg^−1^ in stressed samples.

#### 3.3.3. Shotgun of PM

The abundances of PM-bound redox system-related proteins were analyzed with shotgun proteomics (Table 2, Supplementary Table S5B). Catalases, non-*haem* peroxidases, or dioxygenases were not identified in PM, whereas *Zm*APx05 (B6TM55) was found and showed a 1.73-fold decreased abundance under 24 h of hypoxia. Additionally, *Zm*Prx03, *Zm*Prx24, *Zm*Prx81, and *Zm*Prx85 were detected with increased abundances. In addition, *Zm*PDIL1-1, *Zm*PDIL1-2, and *Zm*PDIL2-1 (A0A1D6PSC4, A0A1D6F5C3, and Q5EUD7), *Zm*LOX1, *Zm*LOX2, *Zm*LOX3, and *Zm*LOX4 (A0A1D6N536, A0A1D6N524, A0A1D6L0J6, and C0P840) were found, of which *Zm*LOX1, *Zm*LOX2, and *Zm*LOX3 showed an enrichment in stressed samples. Furthermore, *Zm*SKU5 (C0PG78), *Zm*DoHcytb561 (C4J5B0), two *Zm*MDHs, and two NAD(P)H-quinone oxidoreductases (*Zm*FQR1, B4FWD0; *Zm*WrbA, B6SPB2) showed an at least 1.5-fold increase in stressed samples. Contrary to this, *Zm*AIR12 was 1.3-fold reduced in stressed samples (A0A1D6KJM7).

Three of the PM-bound RLKs showed a more than 2-fold increased abundance (A0A1D6JEH7, A0A1D6DZ88, and A0A1D6IE45). None of the nucleobase-ascorbate transporters, Casparian strip-like proteins, or aluminum-activated malate transporters were found.

Of the 13 known PIPs in maize, shotgun analysis of root PM determined five PIPs that are enriched after 24 h of hypoxia, with *Zm*PIP2;1 and *Zm*PIP2;4 more than 1.5-fold significantly enriched (Figure 9B). The enriched PIPs were, in order of fold change, *Zm*PIP2;6, *Zm*PIP2;4, *Zm*PIP2;5, *Zm*PIP2;1, and *Zm*PIP2;2. One PIP, *Zm*PIP1;5 was more than 2-fold less abundant after 24 h of hypoxia. The remaining seven PIPs (*Zm*PIP1;1, *Zm*PIP1;2, *Zm*PIP1;3, *Zm*PIP1;4, *Zm*PIP1;6, *Zm*PIP2;3, and *Zm*PIP2;7) were not found in shotgun analysis (Table 2). The averaged LFQ intensities of the controls decreased between the found PIPs as follows: *Zm*PIP1;5 > *Zm*PIP2;1 > *Zm*PIP2;5 > *Zm*PIP2;4 > *Zm*PIP2;6 > *Zm*PIP2;2 and of stressed samples: *Zm*PIP2;5 > *Zm*PIP2;1 > *Zm*PIP1;5 > *Zm*PIP2;4 > *Zm*PIP2;6 > *Zm*PIP2;2 (Table 2).

#### 3.3.4. Shotgun of Soluble Fraction

In the shotgun analysis of the soluble fractions (Supplementary Table S2), *Zm*Cat1, 5 *Zm*APx and 33 *Zm*Prx were identified. *Zm*Cat1, *Zm*APx01, *Zm*APx02, *Zm*APx05, and 18 *Zm*Prx showed a more than 2-fold decreased abundance. Only *Zm*Prx99 showed a 16-fold increase in abundance. In addition, three *Zm*GPx and four cysteine peroxiredoxins with only one showing a 7-fold decreased abundance (*Zm*2CysPrx02, B4FM07) were determined. Three SODs were found, with one (*Zm*SODM4, B4F9H6) showing a 5-fold decreased abundance. Four disulfide isomerases, one thioredoxin (A0A804Q653), and a thioredoxin superfamily protein (C4IZH7), as well as one glutathione reductase (B4FWU6), showed a more than 2-fold decreased abundance. Two DHAR and three MDHAR were found, where the MDHAR showed a more than two-fold decreased abundance. Four of seven found MDHs showed a more than 2-fold increased abundance. In addition, one *Zm*WrbA (B6TFN1) was determined with a 5-fold decreased abundance.

One of the proteins of the L-ascorbate biosynthesis pathway (B4FBC2) and one RLK (A0A804NHW6) were found. The latter showed a strong decrease in abundance.

## 4. Discussion

In the present study, we demonstrated that the oil-based setup induced typical symptoms of flooding-like hypoxia stress in maize roots grown in hydroponics. We showed the induction of AQPs of the PIP2 subgroup and several PM-bound redox systems. Here, we discuss the functions of PM redox systems in root growth and redox homeostasis during hypoxia stress. Finally, the function of hypoxia-induced water channels and their regulation by PM redox systems were reconsidered.

### 4.1. The Oil-Based Setup Induced Typical Hypoxia Stress Symptoms

The efficiency of the oil-based setup for hypoxia stress induction was demonstrated by typical flooding symptoms. For 24 h of hypoxia stress, this includes (i) decreased concentrations of oxygen in the hydroculture medium, (ii) an elevated level of ethylene in maize roots (Figure 2A) corresponding with an upregulated expression of ethylene biosynthesis genes (Supplementary Table S1), (iii) the formation of aerenchyma [19], and (iv) an increased ADH activity (Figure 2B) corresponding with an upregulation of anaerobic genes (Supplementary Table S1). The function of ethylene-induced aerenchyma in the facilitation of gas transport and the expression of anaerobic genes as a switch from aerobic to anaerobic metabolism for ongoing ATP energy production was discussed elsewhere [6]. An elevated level of H_2_O_2_ in maize roots and higher abundances of class III peroxidases with increased activity were determined in PM preparations under hypoxia stress [19].

Gene ontology analysis of RNA-Seq data revealed that metabolic and biosynthetic processes are downregulated on the gene level (Figure 4) probably due to potential energy deficit under low-oxygen conditions. The main biological processes of the DEGs and the PM-bound proteins (“response to oxidative stress”, “hydrogen peroxide catabolic process”, and “plant-type cell wall organization”) confirmed previous results [19] and indicated alterations in additional redox proteins. “Peroxidase activity” or “oxidoreductase activity” as the main molecular function and “*haem*-peroxidase domains” and “LOX domains” correlated well with these findings. Here, an increase in redox activity (Figure 9A) and alterations in abundances of redox proteins at the PM were determined (Table 2). Additionally, “transporter activity” and “water channel activity” as molecular functions and “aquaporin-related domains” on gene and protein levels indicated an alteration in water channels.

### 4.2. Hypoxia-Induced Redox Systems Effected Root Architecture

A function of anaerobic genes and *air12* in lateral root formation was shown [59,60]. The lower transcript level and protein abundance of *Zm*Air12 suggested that its role in lateral root formation may be negligible after 24 h of hypoxia stress (Table 2). The result demonstrated that this protein is transcriptionally regulated. Alterations of *Zm*SKU5 and *Zm*LOX fit with changes in root architecture under hypoxia. SKU5 is involved in directional root growth [61]. Its functions in cell polar expansion and cell wall synthesis have been demonstrated [62]. Cell wall remodeling was supported by upregulation and higher abundances of PM-bound peroxidases under hypoxia. Additionally, upregulation of key enzymes of the phenylpropanoid pathway, higher abundances of dirigent proteins, and a fasciclin-like arabinogalactan were observed [19].

Lipoxygenases inhibit lateral root development [63] but increase the formation of primordia [64]. Lateral root formation seems to be linked to the cooperation of 9-LOX and 13-LOX [64]. Here, *Zm*LOX1, *Zm*LOX2, and *Zm*LOX3 showed increased protein abundances in PM that might be linked to primordia formation under hypoxia (Table 2). The upregulated expressions of *zmlox4*, *zmlox5*, *zmlox8*, and *zmlox10* (Supplementary Table S1) suggested an involvement in ongoing lateral root formation. *Zm*LOX3 has a function in normal plant growth, whereas an overexpression of *zmlox5*, *zmlox8*, and *zmlox10* was shown to compensate for a missing *zmlox3* expression [65]. Here, the upregulation of *zmlox5*, *zmlox8*, and *zmlox10*, accompanied by the *zmlox3* expression and *Zm*LOX3 abundance, correlated with the observed hypoxia-induced adaptations in root growth [66].

Aquaporins accomplish hydraulic conductivity, which is essential for plant growth. Both the inhibition and mutation of AQPs reduced hydraulic conductivity [28,37,67] accompanied with an impaired root growth [68]. Function of *zmpip1;3*, *zmpip2;1*, *zmpip2;2*, *zmpip2;4,* and *zmpip2;6* in root growth has been suggested [69]. Hypoxic and anoxic treatment also reduced hydraulic conductivity [70,71,72]. Under anoxic conditions, the cytosolic pH was decreased which regulates the gating of AQPs [35,36,73]. Contrarily, the overexpression of PIPs (e.g., *scpip1* and *ospip1;2*) increased root growth [74,75]. The lower abundances or the absence of PIP1s in hydroponically grown maize roots (Figure 9B) suggests a function in the development of primary roots. Contrary to this, higher abundances of PIP2 under hypoxia might regulate root hair development in hydroponics to compensate nutrient uptake. The mutation in *atpip2;4* resulted in longer root hairs in the control and phosphate-deficient environment [76]. Additionally, suberization of the endodermis inhibits water flow through the apoplastic pathway. Suberized endo- and exodermal cells were determined in maize root cross-sections under hypoxia [19]. This effect requires AQPs with a high hydraulic conductivity, which was shown for PIP2 isoforms [30,31]. The abundances of *Zm*PIP2;1, *Zm*PIP2;4, *Zm*PIP2;5, and *Zm*PIP2;6 increased under hypoxia (Figure 9B). It was demonstrated that *Zm*PIP2;5 was more abundant in cortical cells of the suberized root hair zone but not in the exodermis, suggesting a role in radial water transport, restricted by the Casparian strip [77,78]. The function of each PIP isoform in specific root cells and under hypoxia stress needs further evaluation.

### 4.3. Hypoxia-Induced PM Redox Systems Are Involved in ROS Production

The upregulation of *zmrboh10* and the observed high levels of H_2_O_2_ confirmed ROS production at the PM under hypoxia stress in maize roots [19]. Thus, the higher abundance of *Zm*PIP2;5 under hypoxia (Table 2) correlated with its function in H_2_O_2_ transport [79]. High levels of ROS caused an imbalance of the cellular redox homeostasis. The RNA-Seq analysis revealed a differential regulation of several redox systems in maize roots under hypoxia (Supplementary Table S1). It appears that the majority of antioxidant genes showed a lack-phase in their response to hypoxia with an upregulation after 48 h [80], possibly as a mechanism to ensure high H_2_O_2_ levels for aerenchyma formation [19]. Genes of major antioxidant systems including those of the Foyer–Halliwell–Asada cycle were not or downregulated after 24 h; the only exceptions were *zmapx04* and the *zmsodm4*. Downregulation of *zmapx*, *zmcat,* and *zmsod* and their decreased abundance in shotgun data of the soluble fraction correlated with the observed lower TMB activity of these fractions compared to controls (Figure 9A). The observed upregulation of mitochondrial SOD and the ubiquinone biosynthetic pathway suggested ROS production by the respiratory chain [81]. The upregulation of the anaerobic pathway makes this suggestion unlikely (Supplementary Table S1). Transcripts of alternative oxidase were not regulated and complex III was downregulated (Supplementary Table S1). The observed upregulation of *zmrboh10* and *zmdiox01* correlated with their function in signal transduction during hypoxia [82,83].

Shotgun proteomics revealed alterations in abundances for several PM-bound redox systems by hypoxia (Table 2). The increased TMB oxidation by PM fractions in the presence of H_2_O_2_ (Figure 9A) suggested higher abundances of *haem* and copper-containing proteins in stressed samples [84,85]. In accordance with this observation, an increase of members of the Cytb561 family (DoHCyt*b*561) and the copper oxidase *Zm*SKU5 were detected in PM under hypoxia (Table 2). The higher abundances of class III peroxidases confirmed the observed upregulation and increased activity in maize root PM under hypoxia [19]. In the shotgun experiment, the abundance of *Zm*Rboh10 was below the limits of detection. Either the transcript was not translated or *Zm*Rboh10 increased only in a few specialized cells for aerenchyma formation, as demonstrated for *Os*RbohH [19,86]. Thus, low-abundant members of the flavocytochrome *b* family (Rboh) and sulfhydryl-groups of membrane proteins may also account for the observed TMB oxidation [87].

Apart from the upregulation of *zmrboh10*, the increased abundance of *Zm*LOX1, *Zm*LOX2, and *Zm*LOX3 supported a production of ROS at the PM. The influence of the various LOX isoforms in oxidative stress-induced lipid peroxidation is still unclear. The higher activity and abundance of *Zm*LOX2 and *Zm*LOX9 together with increased malondialdehyde levels, a marker for lipid peroxidation, was shown for waterlogged maize and pepper leaves overexpressing *calox1* [88,89]. Contrary to this, a lack of *atlox1* and *atlox5* increased the susceptibility to singlet oxygen and led to lipid peroxidation [90]. The association of *Zm*LOX1, *Zm*LOX2, and *Zm*LOX3 with the maize root PM and their enrichment under hypoxia supported the role in lipid peroxidation.

The turnover of glycolytically derived ATP produce protons as a source of intracellular acidosis [91]. Different proton-consuming metabolic pathways have been discussed for maize under hypoxia [92,93]. A higher abundance of the PM H^+^-ATPase was observed in hypoxia stressed samples (Table 2). In addition, cytosolic MDH acts as a biochemical buffer system [94]. Two MDHs showed higher abundances in the PM of stressed samples (Table 2). At least one of these enzymes showed a significant higher affinity for oxaloacetate compared to malate [95]. The consumption of protons by the reaction of PM-bound MDH may be part of the mechanism that counteract against intracellular acidosis [96]. A PM localized malate shuttle involved in pH control was postulated [97]. This hypothesis was supported by the observed differential regulation of five putative PM-bound aluminum-activated malate transporters (ALMT) by hypoxia, with ALMT10 significantly upregulated (Supplemental Table S1).

### 4.4. Hypoxia-Induced PM Redox Systems Are Involved in Membrane Protection and Redox Homeostasis

Oxidative stress caused increased activities of antioxidative systems [5]. The upregulation of *zmapx04*, *zmsodm4* (Supplementary Table S1), and membrane-bound antioxidative systems in hypoxia-stressed samples was observed [19]. A function in membrane protection was already discussed for the PM-bound class III peroxidases *Zm*Prx03, *Zm*Prx24, *Zm*Prx81, and *Zm*Prx85 in maize roots under hypoxia [19]. Although physiological functions of PM redox systems are not completely understood, the data at hand supported a regulatory role in the apoplastic redox homeostasis [45,98]. This was demonstrated for members of the SKU5 similar protein family [99]. Interestingly, the abundance of a *Zm*DoHcytb561 increased measurably under 24 h of hypoxia (Table 2). It was suggested that *At*DoHcyt*b*561 regenerates apoplastic ascorbate by a transmembrane electron transfer and thereby regulates redox homeostasis during oxidative stress [45]. The importance of this reaction becomes obvious by the downregulation of cellular antioxidant systems, ascorbate biosynthesis, and ascorbate transporters during this early phase of hypoxia stress (Table 2, Supplementary Table S1) which was also confirmed by the lower abundances of antioxidative systems in the soluble fractions (Supplementary Table S2).

The molecular mechanism for the maintenance of the apoplastic redox homeostasis under oxidative stress was reflected in the co-regulation of ROS producing PM redox systems (Rboh10 and LOX) and membrane protection by antioxidative systems such as PM-bound class III peroxidases, the DoHcyt*b*561, and SKU5. Additionally, vitamin K_1_ has been identified in maize and soybean PM [44,47] that acts as a mobile electron and proton carrier and as an effective antioxidant inside the membrane [100,101]. The upregulation of *menG* (Supplementary Table S1) and the higher abundance of the NAD(P)H:quinone reductase (Table 2) indicated a de novo synthesis of vitamin K_1_ in root PM [102]. Further, the upregulation of the ubiquinone biosynthetic pathway (Supplementary Table S1) and downregulation of complex III further supported the function of vitamin K_1_ in membrane protection. Both ubiquinone and vitamin K_1_ were demonstrated to act as effective antioxidants [103]. During oxidative stress, NAD(P)H:quinone reductases recycle the vitamin K_1_ pool inside the PM. Vitamin K_1_ may also have the potential to reduce the DoHcyt*b*561. At least a NAD(P)H-dependent cytochrome *b* reduction has been observed by naphthoquinones in maize root PM [44].

### 4.5. Redox Regulation of H_2_O_2_ Transporting Aquaporins

The gene expression of almost all maize root *zmpips* decreased after 24 h of hypoxia (Table 2), which was also determined in hypoxic lateral and adventitious roots [104]. This downregulation might be related to energy deficits caused by the stress. Additionally, the increased ethylene level in maize roots after 24 h of hypoxia (Figure 2A) might lead to reduced *pip* expression, as shown in rose leaves [105]. The missing gene expression of *zmpip2;7* in maize roots is probably associated with a restriction to specific cell types beyond root tissue, as shown by [24] and [106].

Shotgun analysis revealed six *Zm*PIPs with *Zm*PIP2;1, *Zm*PIP2;2, *Zm*PIP2;4, *Zm*PIP2;5, and *Zm*PIP2;6 that increased in abundance after 24 h of hypoxia, two of them significantly (*Zm*PIP2;1 and *Zm*PIP2;4) (Table 2). Other maize root PIPs with no or downregulated gene expression might have a protein abundance below the limits of detection. Interaction partners of AQPs for hetero-tetramers or complexes with other proteins were investigated [26,107,108,109]. On the contrary, a re-localization of *At*PIP2;1 from the PM to subcellular membranes was induced by H_2_O_2_ [110] that revealed higher levels in hypoxia stressed maize roots [19]. The function of PIPs in water transport and, in this regard, the more efficient PIP2 subgroup was sufficiently studied [30,111]. Ethylene was found to be a direct regulator of the *At*PIP’s phosphorylation, leading to enhanced water channel activity [112]. The increased ethylene level (Figure 2A) might serve as an activator of the PIP2 subgroup that showed higher abundances in PM of hypoxia-stressed maize roots. In accordance with these results, upregulation of *Pt*PIP2;4 and *Pt*PIP2;5 has been observed in aspen (*Populus tremuloides*) roots under hypoxia [72]. Anyway, a function in the transport of water and dissolved gases (e.g., oxygen) under hypoxia stress in hydroponics might be significantly reduced. This hypothesis was supported partially by the downregulation of *pip1;1* and *pip1;2* and the absence of the proteins corresponding to these genes in the PM (Table 2). The oxygen deficiency is probably counteracted with aerenchyma; the energy deficit with the switch to anaerobic metabolism.

Due to the similar chemical properties of water and H_2_O_2_, several studies of AQPs examined their function regarding the transport of ROS [79,113,114]. Evidence for a H_2_O_2_ transport was found for *At*PIP2;4, *Zm*PIP2;5, and *Os*PIP2;2 [79,115,116,117]. The structural similarity of *At*PIP2;4 with *Zm*PIP2;1, *Zm*PIP2;2, *Zm*PIP2;4, *Zm*PIP2;5, and *Zm*PIP2;6 (Supplementary Figure S2) suggests a potential role in H_2_O_2_ transport [79]. This hypothesis was further supported by the observation that *At*PIP2;2, *At*PIP2;4, *At*PIP2;5, and *At*PIP2;7 were permeable for H_2_O_2_ in yeast cells and differentially regulated under abiotic stress [115,116].

Additionally, the interaction of *At*DoHCyt*b*561 with *At*PIP1;2 and *At*PIP2;1 has been shown under control and stress conditions [118]. The lower abundance of *Zm*PIP1;2 and the higher abundance of *Zm*PIP2;1 fit well with the higher abundance of *Zm*DoHcyt*b*561 and its postulated function in the regulation of the apoplastic redox homeostasis (Table 2). Besides *Zm*PIPs, class III peroxidases, *Zm*DoHcyt*b*561, Rboh, and other PM redox systems have been detected in detergent-resistant membranes that present functional microdomains [119,120]. For *At*PIP2;1, the interaction with CASPL and members of the RLK family have been observed [118,121]. At least one CASPL was upregulated in maize roots under 24 h of hypoxia (Supplementary Table S1). While CASPL was suggested to direct PIPs to microdomains with specialized functions, RLKs (RKL1 and Feronia) modulate PIP activity [121]. Three members of the RLK family showed higher abundances in maize root PM and two transcripts were upregulated under hypoxia (Supplementary Table S5B). At least RK20-1 has a function in fine-tuning cell growth, controlling apoplastic pH, and ROS production [122,123,124]. Moreover, the lipid composition of microdomains may have an additional effect on the gating of PIPs [125].

An early idea on the function of PM redox was the regulation of SH-groups of trans-membrane proteins, e.g., transporters [42]. Currently, the occurrence of vitamin K_1_ in PM is generally accepted and quinone perception via Leucin-rich repeat RLK has been demonstrated [44,47,126]. Vitamin K_1_ directly effects the redox status of SH-groups and serves as an electron carrier for protein disulfide bond formation [127,128,129,130]. The later reaction is important for the assembly of protein complexes and redox regulation [131]. Further, H_2_O_2_ can also cause intermolecular disulfide bonds [132,133].

Redox regulation of maize AQPs was suggested by [38], who observed an oxidative gating of water channels in maize roots by H_2_O_2_. Amino acid sequences of maize PIPs were highly conserved with three to four Cys residues (Supplementary Figure S3). For *Zm*PIP2;5, a conserved disulfide bond was demonstrated for Cys-79 between two monomers in loop A [134]. It was shown that Cys-79 was not a target of redox regulation [109]. Redox regulation, as found in AQP8 [39], may be also excluded, because the Cys-53 residue was not conserved in *Zm*PIPs (Supplementary Figure S3).

The prediction of the putative structure of *Zm*PIP2;5 and *Zm*PIP2;6 revealed a conserved Cys residue at transmembrane helix III that was oriented to the lipid bilayer (Supplementary Figures S2 and S3). A comparable Cys residue has been shown in *So*PIP2;1 by crystallography [35]. The higher level of H_2_O_2_ [19] and the observed lipid peroxidation in maize roots by hypoxia [135] may cause oxidation of those SH-groups in *Zm*PIP2;1, *Zm*PIP2;2 *Zm*PIP2;4; *Zm*PIP2;5, or *Zm*PIP2;6 to sulfinic, sulfenic, or sulfonic acid groups. Vitamin K_1_, as an antioxidant acting inside the PM, can keep those SH-groups reduced.

### 4.6. Transcriptome to Proteome Regulation

After 24 h of hypoxia, transcript and protein abundance at the PM were mainly negatively correlated with a decreased gene expression and an increased protein abundance. A transcriptional positive regulation was indicated for 9.5% of the proteins listed in Table 2 including Air12, WrbA, Prx85, and the PIP1;5. A decrease in *pip* expression and PIP accumulation was observed under different stress conditions [136,137]. Nonetheless, gene expression usually proceeds early after stress induction. Therefore, genes with a negative correlation were selected and analyzed by RT-qPCR at earlier time points (Figure 6). The results after 6 and 12 h confirmed the downregulation of those genes by hypoxia. To explain the negative correlation, a deeper look into mRNA-to-protein translation processes is necessary [138,139,140]. Some data presented an increased protein abundance with no alteration in its gene expression even under stress [141,142]. In addition, a correlation between mRNA expression and protein product depends on significance [143]. A difference between RNA-Seq and RT-qPCR was observed for smaller genes with fewer exons and lower expression [144], which was demonstrated for the regulation of the PM-bound class III peroxidases under hypoxia stress [19]. In addition, major post-transcriptional regulations during hypoxia were suggested. About 40% of the maize genome were intron-containing genes that undergo alternative splicing [145]. This post-transcriptional modification has been demonstrated at least for PIP2;7 under salt stress [146]. Besides, alternative splicing regulates the targeting of MDH [147]. Altogether, post-transcriptional protein regulation under stress is still a field that needs broader attention. For the MDHs (Table 2), the predicted S-palmitoylation may guide the proteins to the PM. Other post-translational modifications, e.g., ubiquitination, alter the abundances of proteins via proteasomal degradation, which was shown for Na^+^/K^+^-ATPase under hypoxia in eukaryotic cell lines [148] and Arabidopsis root microsomes under osmotic stress [149]. An effect of hypoxia on decreased protein degradation also seems possible. Additionally, an alternative protein translation pathway involving hypoxia-induced transcription factors was discussed [138,150,151].

## 5. Conclusions

In the present study, an oil-based setup was established to analyze hypoxia-induced changes in maize roots. A combination of transcriptomics and proteomics revealed general molecular mechanisms in hypoxia-response and proteins at the PM that effect root architecture and maintain redox homeostasis under oxidative stress. Proteins involved in ROS production were either upregulated (*zmrboh10*) or accumulated (*Zm*LOX1, *Zm*LOX2, and *Zm*LOX3) in stressed samples. The simultaneous induction of the PIP2 subgroup supported H_2_O_2_ transport across the PM, whereas induction of PM redox systems (Prx, DoHcyt*b*561, NAD(P)H:quinone reductase) increased ROS scavenging, recycled apoplastic ascorbate and the vitamin K_1_ pool that act as an antioxidant inside the lipid bilayer. The observed upregulation of vitamin K_1_ biosynthesis supported this suggestion. Redox regulation of PIPs will need future elucidation.

## Figures and Tables

**Table 1:** Primer sequences in 5′-3′-orientation for RT-qPCR.

Gene ID	Name	Sequence forward Primer 5′-3′	Sequence Reverse Primer 5′-3′
542619	PIP2-5	ACTGGATCTTCTGGGTGGGT	CGATCTAGCGGCTGAAGGAG
541888	PIP2-1	CACTGGATCTTCTGGGTGGG	GATGGCATTCTCCTCGCTCAC
542644	PIP2-2	TCGATCTAGCGTGGGGAGAG	AACAAAAGCGACCGACGAGA
100285512	DoHCyt*b*561	GCCGTTGTTCAGAGAGACAT	AGGAGTACAGACTACAGAGGC
100383470	SKU5	TCTACTTCCCACCCCTTGGT	AGGTGCGTGTGGTTCATCTT
100285365	FQR1	GGGTTCAGCCTGATCTACACAT	GAGCCACACAACATCCAGAC
541856	LOX1	TACACGCTGCTCTACCCCAA	CCACACTTCACGGAACGGAA
100037802	LOX2	GCCAGCGTTTCACCCAAAAA	CCAGCACCAGTACACCAAGG
542495	LOX3	CAGCCTCACACAGACACCAA	GATGATCCCGCTCAGCATCT
100273405	EF-TuM	CGCAGTTGATGAGTACATCC	AACACGCCCAGTAACAACAG

**Table 2:** Alterations of PM redox systems, ascorbate transporters, and PIPs on transcript and protein level after 24 h of hypoxia stress. Nomenclature of peroxidases according to RedoxiBase (Available online: https://peroxibase.toulouse.inra.fr/ (accessed on 4 May 2019)) [58]. Significant differences between stressed and controls, calculated with Student’s *t*-test, were marked at *p* < 0.001 (***), *p* < 0.01 (**), and *p* < 0.05 (*). fc, fold change; accession number, Acc; MW, molecular weight in kDa; pI, isoelectric point; TMH, transmembrane helices; GPI, glycosylphosphatidylinositol anchor; DoHcyt*b*561, Cytochrome *b*561, and DOMON domain-containing protein; MDHAR, Monodehydroascorbate reductase homolog 1.

Protein Name	Gene ID	Gene fc	UniProt Acc.	Protein fc	MW ^(1)^	pI ^(1)^	TMH ^(2)^
Auxin induced in root cell cultures, AIR12	100280845	−1.95 *	B6SN55	−1.30	25.1	9.39	0/GPI
Auxin induced in root cell cultures, AIR12	100285927	−4.05 *	B6UBU0	n.d.	26.7	9.34	0/GPI
Auxin induced in root cell cultures, AIR12	103653191	−1.33	A0A3L6EZL2	n.d.	24.0	9.39	0/GPI
Auxin induced in root cell cultures, AIR12	100286361	−1.88 *	K7VGM8	n.d.	22.2	6.90	0/GPI
DoHcyt*b*561	100285512	−1.17	B6U5U8	2.78	41.0	9.57	5
H^+^-ATPase	542052	−1.03	K7TX67	1.41	104.9	6.23	8
H^+^-ATPase	100502231	−2.14	A0A1D6MV33	1.03	105.4	6.20	8
H^+^-ATPase	542048	−1.75 *	A0A1D6DVJ7	1.78	81.0	5.68	5
Lipoxygenase 2, LOX1	541856	−1.82	Q9LKL4	2.17	98.2	6.25	0
Linoleate 9S-lipoxygenase 2, LOX2	100037802	−4.09	A1XCH8	1.99	98.4	6.17	0
Lipoxygenase, LOX3	542495	1.33	Q8W0V2	2.03	96.5	5.72	0
Lipoxygenase, LOX4	100037803	3.38 *	C0P840	−1.06	100.4	6.18	0
Malate dehydrogenase, MDH	542598	−1.18	Q08062	2.33 *	35.6	5.75	0
Malate dehydrogenase, cMDH	100280767	−1.42 *	A0A1D6GPH0	1.73	33.3	7.00	0
MDHAR	100501585	−1.30 **	C4J4E4	n.d.	46.7	5.45	0
Monocopper oxidase-like protein, SKU5	100383470	−2.08 **	C0PG78	1.49	65.8	6.01	1
NAD(P)H dehydrogenase (quinone), FQR1	100285365	−1.55 *	B6U474	2.36	21.5	6.06	0
NAD(P)H dehydrogenase (quinone), WrbA	100280914	1.17	B6SPB2	1.44	25.7	6.98	0
Peroxidase 3	542505	−2.27	A0A1D6LYW3	3.18	38.7	6.52	0
Peroxidase 24	542464	−1.17	B4FHG3	4.72	37.9	5.51	0
Peroxidase 81	100193733	−2.89	B4FG39	12.57 *	36.6	8.08	1
Peroxidase 85	100279351	1.53 *	A0A1D6E530	5.74	35.5	5.35	1
Respiratory burst oxidase 10, Rboh10	100381459	5.91	C0HG64	n.d.	45.2	9.22	2
Nucleobase-ascorbate transporter 6	100272944	−1.21	B4F8S3	n.d.	57.7	9.37	11
Nucleobase-ascorbate transporter 6	100282103	1.48	C0PH14	n.d.	58.1	9.31	11
Permease I	100283406	−1.39	B4FIZ3	n.d.	57.4	9.54	11
Nucleobase-ascorbate transporter 2	103652969	−1.14	K7U0A9	n.d.	57.3	8.65	9
Nucleobase-ascorbate transporter 3	100279038	1.30	A0A096QFX2	n.d.	60.5	9.13	11
Nucleobase-ascorbate transporter 2	100279209	1.14	A0A1D6EP29	n.d.	76.6	9.60	8
Nucleobase-ascorbate transporter 12	100279219	−1.34	A0A1D6G835	n.d.	77.8	9.33	10
PIP1;1 active with PIP1;2	542434	−3.21 *	Q41870	n.d.	30.9	9.47	6
PIP1;2 active with PIP1;1, PIP2;1, PIP2;4, or PIP2;5	541779	−4.68 ***	Q9XF59	n.d.	30.8	9.00	6
PIP1;3/1;4	541886	−2.22 *	Q9AQU5	n.d.	31.0	8.83	6
PIP1;5	542014	−2.65	Q9AR14	−2.06	30.7	8.30	6
PIP1;6	113523644	n.d.	Q9ATN0	n.d.	31.0	6.70	6
PIP2;1 active with PIP1;2	541888	−1.94	Q84RL7	1.54 *	30.2	7.69	6
PIP2;2	542644	−1.92	Q9ATM8	1.23	30.3	8.29	6
PIP2;3	541889	−2.55 *	Q9ATM7	n.d.	30.4	6.95	6
PIP2;4 active with PIP1;2	541890	−3.65 *	Q9ATM6	1.75 *	30.3	6.50	6
PIP2;5 active with PIP1;2, impaired by Hg^2+^	542619	−4.29 **	Q9XF58	1.69	29.8	7.70	6
PIP2;6	541891	−2.12 **	Q9ATM5	2.39	30.2	8.38	7
PIP2;7	542645	n.d.	Q9ATM4	n.d.	30.8	8.26	6

^(1) + (2)^ see methods.

## Data Availability

Data are available via ProteomeXchange with identifiers PXD028987 (PM) and PXD033386 (soluble fraction).

## Supplementary Materials

The following supporting information can be downloaded at: https://www.mdpi.com/article/10.3390/antiox11050836/s1. Table S1: RNA-Seq analyses; Table S2: Overview of selected shotgun and RNA-Seq data of 24 h hypoxia-stressed maize root soluble proteins; Table S3: Quantifying mapping results of DAVID; Table S4: Gene ontology enrichment analysis of differentially expressed genes using DAVID; Table S5: Overview of selected shotgun and RNA-Seq data of 24 h hypoxia-stressed maize root plasma membrane proteins; Table S6: Gene ontology enrichment analysis of PM proteins using DAVID; Figure S1: Quality control of RNA isolation and RNA-Seq data; Figure S2: CLUSTAL O (1.2.4.) multiple sequence alignment of PIPs; Figure S3: Hypothetical model of *Zm*PIP2;5. Reference [152] are cited in the supplementary materials.
