# Supplementary material for: Hypoxia-Induced Aquaporins and Regulation of Redox Homeostasis by a Trans-Plasma Membrane Electron Transport System in Maize Roots
Source: Antioxidants (Basel). 2022 Apr 25;11(5):836. doi: 10.3390/antiox11050836 (PMC9137787; doi:10.3390/antiox11050836)
Supplement: Supplementary file 1 [file antioxidants-11-00836-s001.zip › Supplementary data.docx.pdf]

antioxidants

MDPI

Supplementary data

Hypoxia-induced aquaporins and regulation of redox homeostasis by a trans-plasma membrane electron transport chain in maize roots

Anne Hofmann [1], Stefanie Wienkoop [2] and Sabine Lüthje [1,*]

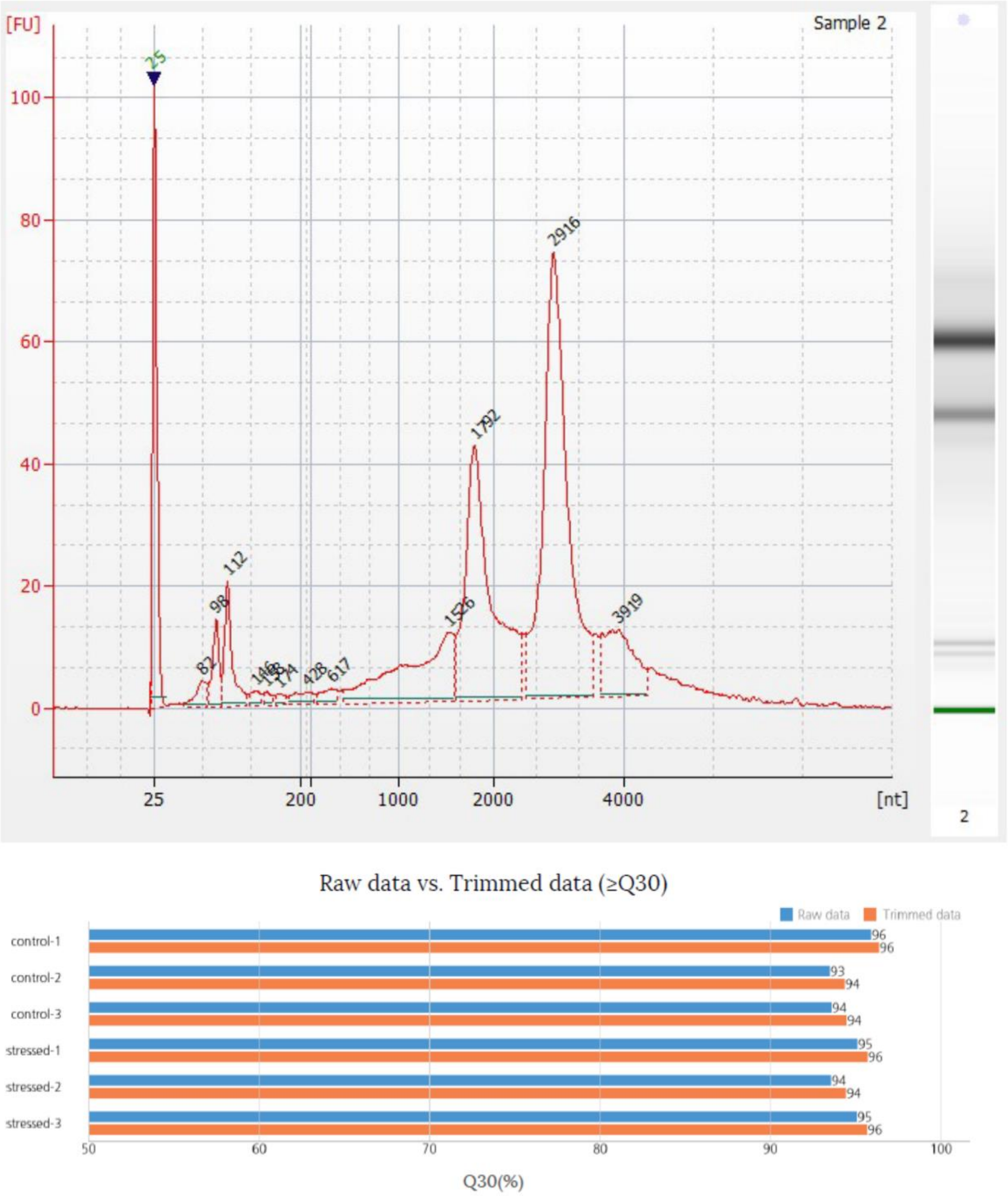

Figure S1. Quality control of RNA isolation and RNA-Seq data. Quality control of a representative RNA sample analysed with Bioanalyzer RNA Pico 6000 chip. In detail, Macrogen quantifies the starting genomic material by a fluorescence-based quantification done by Picogreen (Invitrogen, Waltham, USA) method using Victor 3 fluorometry. The company checked total RNA integrity using an Agilent Technologies 2100 Bioanalyzer. Only RNA samples with an RNA Integrity Number (RIN) value greater than or equal to 7 were used for RNA Sequencing. Gel electrophoresis, shown on the right, was used to reveal the condition (including the presence or absence) of DNA in the samples.

The Phred quality score measures the quality of the identification of the nucleobases generated by automated DNA sequencing. Here, this Q30 score (in %) is equivalent to the probaility of 1 incorrect base call in 1000 times.

```
AtPIP2;4    ---------MAK---D---LDVNESGPPAARDYKDPPPAPFFDMEELRKWPLYRAVIAEF  45
ZmPIP1;5    MEGKEEDVRLGANRYSERQPIGTAAQGTEEKDYKEPPPAPLFEAEELTSWSFYRAGIAEF  60
ZmPIP2;1    ---------MGK---DDVIESGAGGGEFAAKDYTDPPPAPLIDAAELGSWSLYRAVIAEF  48
ZmPIP2;2    ---------MGK---DDVVQSGAGGGEFAAKDYTDPPPAPLVDAAELGSWSLYRAVIAEF  48
ZmPIP2;4    ---------MAK----DIEASGPEAGEFSAKDYTDPPPAPLIDAEELTQWSLYRAVIAEF  47
ZmPIP2;5    ---------MAK----DIEAAA----AHEGKDYSDPPPAPLVDAEELTKWSLYRAVIAEF  43
ZmPIP2;6    ---------MGK----EVDVSTLEAGGVRDRDYADPPPAPLIDIDELGKWSLYRAVIAEF  47
                     :.                   :** :*****:.:  ** .* :*** ****

                                       Loop A
AtPIP2;4    VATLLFLYVSILTVIGYKAQTDATAGG----VDCGGVGILGIAWAFGGMIFVLVYCTAGI  101
ZmPIP1;5    VATFLFLYISILTVMGVSKS----------SSKCATVGIQGIAWSFGGMIFALVYCTAGI  110
ZmPIP2;1    IATLLFLYITVATVIGYKHQTDASAS--GADAACGGVGVLGIAWAFGGMIFVLVYCTAGI  106
ZmPIP2;2    IATLLFLYVTVATVIGYKHQTDASASGAGADAACGGVGVLGIAWAFGGMIFVLVYCTAGI  108
ZmPIP2;4    IATLLFLYITVATVIGYKHQTDASAS--GPDAACGGVGILGIAWAFGGMIFILVYCTAGI  105
ZmPIP2;5    VATLLFLYITVATVIGYKHQTDAAAS--GPDAACGGVGVLGIAWAFGGMIFILVYCTAGV  101
ZmPIP2;6    VATLLFLYITVATVIGYKHQTDASAS--GPDAACSGVGILGIAWAFGGMIFILVYCTAGI  105
            :**:****::: **:*  .  .             *. **: ****:****** *******:

                       Loop B                                Loop C
AtPIP2;4    SGGHINPAVTVGLFLARKVSLVRTVLYIVAQCLGAICGCGFVKAFQSSYYTRYGGGANEL  161
ZmPIP1;5    SGGHINPAVTFGLFLARKLSLTRALFYMVMQCLGAICGAGVVKGFQEGLYMGAGGGANAV  170
ZmPIP2;1    SGGHINPAVTFGLFLARKVSLVRALLYIVAQCLGAICGVGLVKAFQSAYFDRYGGGANSL  166
ZmPIP2;2    SGGHINPAVTFGLFLARKVSLVRALLYMVAQCLGAVCGVGLVKAFQSAYFDRYGGGANSL  168
ZmPIP2;4    SGGHINPAVTFGLFLARKVSLVRALLYIIAQCLGAICGVGLVKGFQSAYYVRYGGGANEL  165
ZmPIP2;5    SGGHINPAVTFGLFLARKVSLVRALLYIVAQCLGAICGVGLVKGFQSAFYVRYGGGANEL  161
ZmPIP2;6    SGGHINPAVTFGLFLARKVSLVRALLYMAAQSLGAICGVALVKGFQSGFYARYGGGANEV  165
            **********.*******:**.*:::*:  *.***:** ..**.**.. :   ***** :

                                         Loop D
AtPIP2;4    ADGYNKGTGLGAEIIGTFVLVYTVFSATDPKRNARDSHVPVLAPLPIGFAVFMVHLATIP  221
ZmPIP1;5    NPGYTKGDGLGAEIVGTFVLVYTVFSATDAKRSARDSHVPILAPLPIGFAVFLVHLATIP  230
ZmPIP2;1    ASGYSRGTGLGAEIIGTFVLVYTVFSATDPKRNARDSHVPVLAPLPIGFAVFMVHLATIP  226
ZmPIP2;2    ASGYSRGAGLGAEIVGTFVLVYTVFSATDPKRNARDSHVPVLAPLPIGFAVFMVHLATIP  228
ZmPIP2;4    SDGYSKGTGLAAEIIGTFVLVYTVFSATDPKRSARDSHVPVLAPLPIGFAVFMVHLATIP  225
ZmPIP2;5    SAGYSKGTGLAAEIIGTFVLVYTVFSATDPKRNARDSHVPVLAPLPIGFAVFMVHLATIP  221
ZmPIP2;6    SAGYSTGTGLAAEIIGTFVLVYTVFSATDPKRNARDSHVPVLAPLPIGFAVFMVHLATIP  225
              **. * **.***:************** **.*******:***********:*******

                    Loop E
AtPIP2;4    ITGTGINPARSFGAAVIYNNEKAWDDQWIFWVGPMIGAAAAAFYHQFILRAAAIKALGSF  281
ZmPIP1;5    ITGTGINPARSLGAAIVYNRSHAWNDHWIFWVGPFIGAALAAIYHVVIIRALPFKSRD--  288
ZmPIP2;1    VTGTGINPARSLGAAVIYNKDKPWDDHWIFWVGPLVGAAIAAFYHQYILRAGAIKALGSF  286
ZmPIP2;2    VTGTGINPARSLGAAVVYNKDKPWDDHWIFWVGPLLGAAIAAFYHQYILRAGAIKALGSF  288
ZmPIP2;4    ITGTGINPARSLGAAVIYNKDKAWDDQWIFWVGPLIGAAIAAAYHQYVLRASAT-KLGSY  284
ZmPIP2;5    ITGTGINPARSLGAAVIYNNDKAWDDHWIFWVGPFIGAAIAAAYHQYVLRASAA-KLGSS  280
ZmPIP2;6    ITGTGINPARSLGAAVVYNNSKAWSDQWIFWVGPFIGAAIAALYHQIVLRASAR-GYGSF  284
            :**********:***::**..: *.*:*******::*** ** **   ::**      .

AtPIP2;4    GSFGSFRSFA  291
ZmPIP1;5    ----------  288
ZmPIP2;1    RSNA------  290
ZmPIP2;2    RSNA------  292
ZmPIP2;4    RSNA------  288
ZmPIP2;5    ASFSR-----  285
ZmPIP2;6    RSNA------  288
```

Figure S2. CLUSTAL O (1.2.4) multiple sequence alignment of PIPs. Alignment of *At*PIP2;4 (Q9FF53), *Zm*PIP1;5 (GRMZM2G081843), *Zm*PIP2;1 (GRMZM2G014914), *Zm*PIP2;2 (GRMZM2G092125), *Zm*PIP2;4 (GRMZM2G154628), *Zm*PIP2;5 (GRMZM2G178693), and *Zm*PIP2;6 (GRMZM2G047368). Indicated are loops A, B, C, and D (cyan), the four residues involved in the selectivity filter of the channel (yellow), the NPA motif (green), and cysteine residues (blue). Sequence of *At*PIP2;4 was taken from [112].

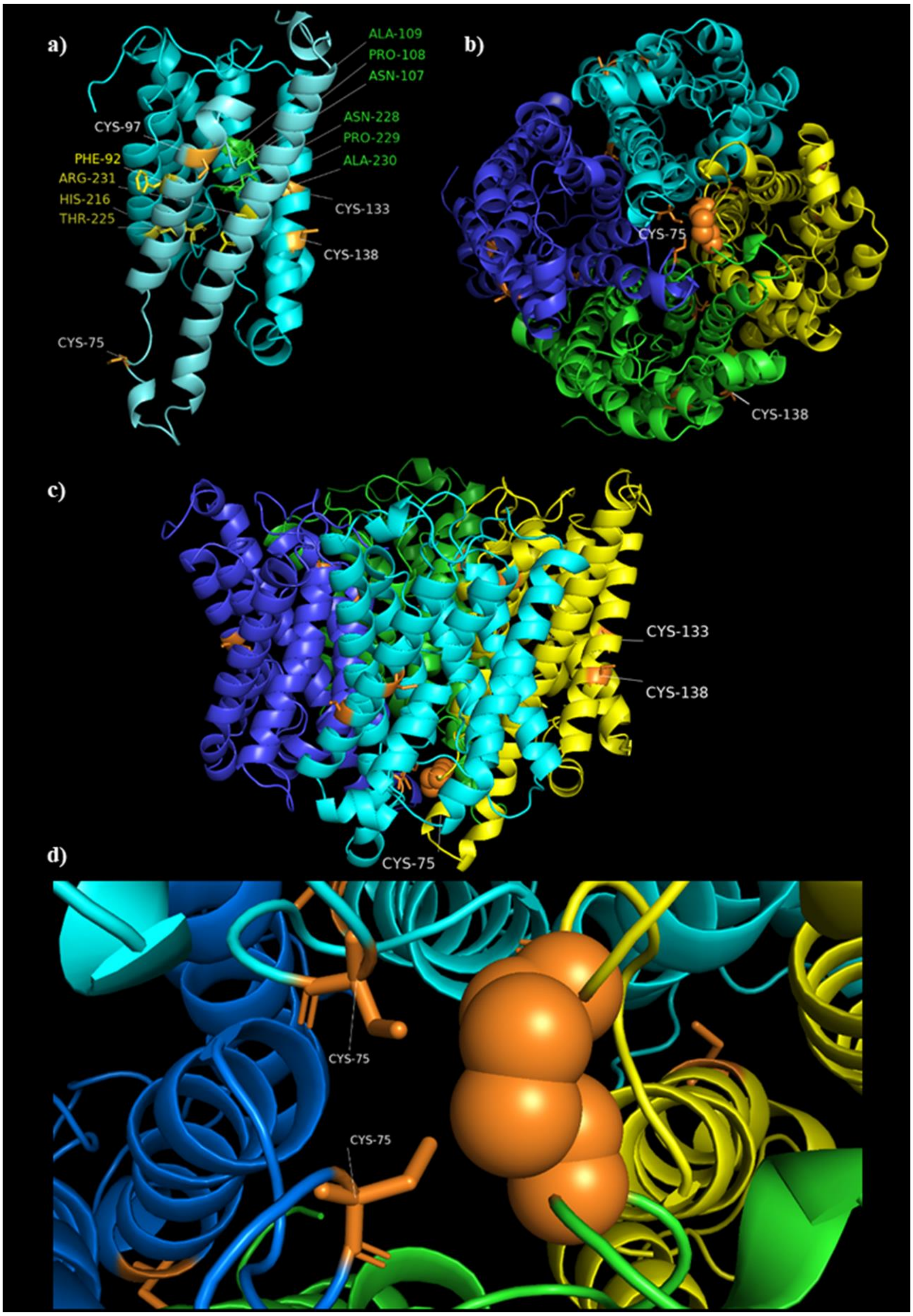

Figure S3. Hypothetical model of *Zm*PIP2;5. Tertiary structure of *Zm*PIP2;5 was modelled by Swiss-Model using spinach (*Spinacia oleracea* L.) aquaporin *So*PIP2;1 (4jc6.2.A) as the template [152]. Shown are **a)** the monomer with the two NPA motifs (green), the four residues involved in the selectivity filter of the channel (yellow) and the four cysteine residues, **b)** the homo tetramer top view and **c)** side view, and **d)** the disulphide bond (spheres, orange) between two Cys-75 of two monomers inside the homo tetramer. Monomers (green, yellow, cyan and marine), cysteine (orange). Visualisation of the hypothetical tertiary structure by PyMOL vers. 2.4.1
